# Assessing average somatic CAG repeat instability at the protein level

**DOI:** 10.1038/s41598-019-55202-x

**Published:** 2019-12-16

**Authors:** Hubert Aviolat, Ricardo Mouro Pinto, Elizabeth Godschall, Ryan Murtha, Hannah E. Richey, Ellen Sapp, Petr Vodicka, Vanessa C. Wheeler, Kimberly B. Kegel-Gleason, Marian DiFiglia

**Affiliations:** 1 Department of Neurology, MassGeneral Institute for Neurodegenerative Disease, Massachusetts General Hospital, Harvard Medical School, Boston, MA USA; 2 Department of Neurology, Center for Genomic Medicine, Massachusetts General Hospital, Harvard Medical School, Boston, MA USA; 3 Present Address: Research Center PIGMOD, Institute of Animal Physiology and Genetics of the Czech Academy of Sciences, Libechov, Czech Republic

**Keywords:** Genetics of the nervous system, Huntington's disease

## Abstract

Sandwich ELISA-based methods use Abs that target the expanded polyglutamine (polyQ) tract to quantify mutant huntingtin (mHTT). Using Meso Scale Discovery (MSD) assay, the mHTT signal detected with MW1 Ab correlated with polyQ length and doubled with a difference of only 7 glutamine residues between equivalent amounts of purified mHTTexon1 proteins. Similar polyQ length-dependent effects on MSD signals were confirmed using endogenous full length mHTT from brains of Huntington’s disease (HD) knock-in (KI) mice. We used this avidity bias to devise a method to assess average CAG repeat instability at the protein level in a mixed population of HTT proteins present in tissues. Signal detected for average polyQ length quantification at the protein level by our method exhibited a strong correlation with average CAG repeat length at the genomic DNA level determined by PCR method in striatal tissue homogenates from *Hdh*^*Q140*^ KI mice and in human HD postmortem cortex. This work establishes that CAG repeat instability in mutant HTT is reflected at the protein level.

## Introduction

Huntington’s disease (HD) is a neurodegenerative disease due to a CAG trinucleotide repeat expansion^1^, ranging from 36 to 250 repeats^2^ and resulting in an extended polyglutamine (polyQ) tract within huntingtin (HTT) protein. Age at disease onset, usually between 30 and 55 years, is strongly and inversely correlated with the size of the expanded CAG repeat^3^ but only explains ~60–70% of the variance in age at onset^3–5^. An early onset of symptoms, before age 20 years, is considered to be the juvenile form of the disease (JHD). JHD patients account for 5–10% of individuals with HD and usually have more than 60 CAG repeats^6^. The inherited expanded CAG repeat is unstable and undergoes a progressive increase in length over time in somatic cells^7–9^. Quantification of somatic CAG repeat instability by PCR in several HD knock-in (KI) mouse models has revealed an initial CAG repeat size, age and tissue dependency of this phenomenon^9–11^. Strikingly, genomic DNA (gDNA) from postmortem brain samples from two HD individuals, who died of other causes and with no microscopic evidence of pathological cell loss in the striatum (inherited CAG repeat length of 41 and 51 and an age at death of 40 and 27 years respectively), showed dramatic mutation length increases in striatum (up to >1,000 CAG repeats) and in the cortex, though to a lesser extent^11^. These observations suggest that somatic instability could precede and influence the onset of symptoms. Small-pool PCR (SP-PCR) analysis of gDNA from postmortem cortical brain tissue in patients with HD also suggested that somatic CAG repeat instability influences age of disease onset, with larger gains in repeat length associated with earlier disease onset^12^. Several candidate genes involved in DNA mismatch repair were identified to drive somatic instability in a mouse model of HD^13,14^. Most notably, GWAS found that the length of the uninterrupted CAG tract drives HD onset in humans, and that polymorphic variation in a region containing DNA repair genes was associated with disease onset or progression of HD, together consistent with somatic CAG expansion as a driver of HD pathogenesis^15–18^.

Technologies allowing quantification of mutant HTT (mHTT) protein are of prime interest, not only for pharmacodynamics, but also for biomarkers of disease evolution. Indeed, using a micro bead-based IP-flow cytometry and Single Molecule Counting (SMC) assay, mHTT in cerebrospinal fluid (CSF) was shown to correlate with HD progression^19,20^. Furthermore, using the SMC assay, dose-dependent reductions of mHTT protein in CSF taken from patients receiving an anti-sense oligonucleotide to lower HTT were reported^21^. Other sandwich ELISA-based assays, such as those based on Meso Scale Discovery (MSD) technology, were also developed for mHTT detection^22^. All these assays are similar and only diverge by the signal read-out that allows for improved assay sensitivity, significantly extending the linear dynamic range beyond that achievable with traditional sandwich ELISA assays read-outs.

Sandwich ELISA-based methods for mHTT quantification use polyQ targeting detection Ab MW1^19,20,22^. While MW1 and other polyQ targeting Abs (1C2 and 3B5H10) were initially proposed to recognize a specific mutant conformation^23,24^ or a specific toxic monomeric conformation^25^, recent studies have contradicted these hypotheses, suggesting a linear lattice model^26–30^. These Abs bind a small polyQ epitope in similar linear and extended conformations, with a higher avidity for expanded polyQ tracts due to the Ab’s bivalence. Thus, these polyQ-binding Abs do not specifically, but preferentially recognize mHTT.

Although the intensity of the signal of sandwich ELISA-based assays for mHTT was reported to be dependent on the polyQ length^19,20,22,31^, no study has accurately quantified this phenomenon. Even when the effects of polyQ length on mHTT quantification were considered, protein concentration was proposed to be the overwhelming contributor in the polyQ range seen in 93% of patients^20,32^. However, this hypothesis was based on results obtained at a single concentration with a different assay, the time-resolved Förster resonance energy transfer (TR-FRET) immunoassay^33^. Moreover, assessment of confounding variables for mHTT quantification in HD patients has revealed an association with inherited CAG repeat length^19,33–35^.

In this study, we assessed the effect of polyQ length on mHTT detection using MSD assay and polyQ targeting Abs. We observed that the signal detected can be as much as double for a variation of only 7 glutamines in the polyQ length range seen in the adult HD patient population^32^. We have taken advantage of this bias to design and validate a novel method to assess mean CAG repeat length at the protein level. This method could become a new benchmark to complement the PCR method, for detection of somatic expansion of unstable CAG repeats at the protein level.

## Results

### PolyQ length in mHTT affects its quantification by MSD assay using polyQ targeting Abs

**Figure 1:**
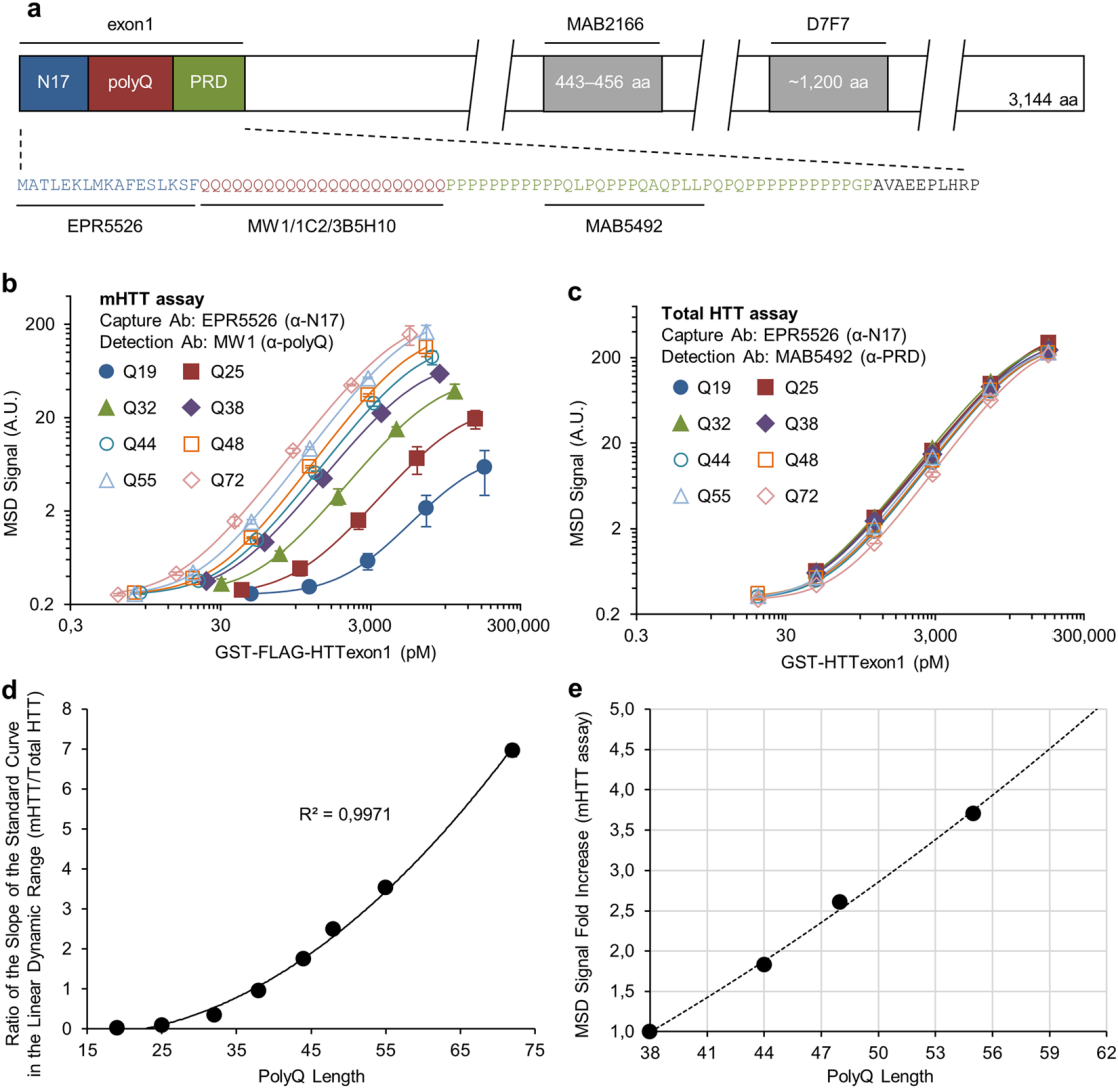
PolyQ length affects GST-FLAG-HTTexon1 quantification by MSD assay using polyQ targeting detection Ab. (**a**) Diagram shows antibody epitopes in human HTT protein (NCBI reference sequence: NP_002102.4). Calibration curve performance for GST-FLAG-HTTexon1 protein using MW1 (**b**) and MAB5492 (**c**) detection Abs. Curves were fitted with a four-parameter logistic regression model with 1/Y^2^ weighting. Mean values ± SD (1 σ) of duplicates of a single experiment are shown. (**d**) Plot of ratio of the slopes determined from standard curves in the linear dynamic range for mHTT assay by total HTT assay as a function of polyQ length exhibits a strong correlation. Mean values ± propagated SD (1σ) of duplicates of a single experiment are shown. (**e**) Using the polyQ length-dependent correlations shown in (**d**), MSD signal fold increase as a function of polyQ length at constant amount of mHTT protein was extrapolated for mHTT assay. mHTT signal predicted for GST-FLAG-HTTexon1 proteins from Q38 to Q62 was normalized by the MSD signal for GST-FLAG-HTTexon1-Q38. PolyQ lengths ranging from Q38 to Q62 correspond to the polyQ length range seen in adult HD patients. GST: glutathione S-transferase; N17: HTT first 17 aa; PRD: proline-rich domain.

The effect of polyQ length on the detection of mHTT by MSD assay was evaluated with a series of purified GST-FLAG-HTTexon1 fusion proteins containing polyQ lengths from Q19 to Q72 (Supplementary Fig. S1a). MSD is a method similar to ELISA except that electrochemiluminescence is used as detection readout: electricity is applied to the plate electrodes leading to light emission by electrochemiluminescent labels that are conjugated to detection antibodies. The monoclonal rabbit capture Ab EPR5526 was paired with different mouse monoclonal polyQ targeting detection Abs MW1, 1C2 and 3B5H10 for mHTT assays (Fig. 1a). A rigorous protocol was developed to achieve the most accurate protein concentrations of the GST-FLAG-HTTexon1 proteins used in the assay (see Methods, Supplementary Figs. S1, S2 and Supplementary Table S1). Results showed that the intensity of MSD signal obtained with MW1 detection Ab increased with increasing polyQ length (Fig. 1b), confirming previous published results. In contrast, the MSD signal intensity seen with the mouse monoclonal MAB5492 detection Ab, a non-polyQ targeting Ab^36^ (Fig. 1a) was solely dependent on protein concentration (Fig. 1c). If used with biological sample, the Abs pair EPR5526-MAB5492 will allow total HTT (WT and mutant) detection. When the slopes of the standard curves in the linear dynamic range obtained by MW1 were normalized by the slopes of the standard curves in the linear dynamic range obtained by MAB5492 (see Supplementary Data Set 1 for Method details), corresponding to mHTT/Total HTT assay, a strong polyQ length correlation was observed (R^2^ = 0.9971; Fig. 1d). Similar correlations were obtained with 1C2 and 3B5H10 detection Abs (R^2^ > 0.98; Supplementary Fig. S3).

To quantify Q-dependent signal rate change observed with MW1 for polyQ lengths in the range of adult HD patients, we extrapolated, from the correlation in Fig. 1d, the mHTT signal fold increase for each additional glutamine residue in GST-FLAG-HTTexon1 protein at constant protein concentration. In this aim, mHTT signal predicted for GST-FLAG-HTTexon1 proteins from Q38 to Q62 was normalized by the MSD signal for GST-FLAG-HTTexon1-Q38. Results showed that predicted mHTT signal with MW1 doubled with the addition of only 7 glutamine residues (Fig. 1e). These results suggest that polyQ length dependent bias has a significant effect on mHTT detection, even for CAG repeats in the *HTT* gene in the pathological range of most HD patients. Other polyQ targeting Abs 1C2 and 3B5H10 also exhibited a polyQ length-dependent bias but to a much lower extent than MW1 (Supplementary Fig. S3).

**Figure 2:**
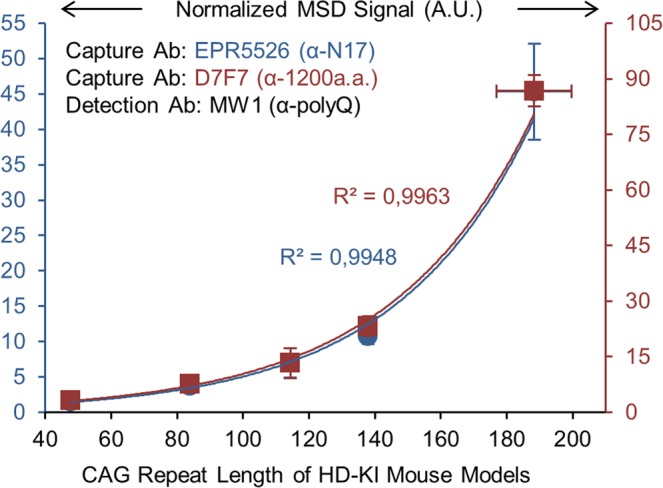
PolyQ length-dependent effect on mHTT detection is also observed with full length mHTT from HD-KI mice. Homogenates from striatum of 6 months old HD-KI mice with 50, 80, 111, 140 and 175 CAG repeats were analyzed for detection of mHTT with two different capture Abs (EPR5526 and D7F7) and MW1 detection Ab. MSD signals were normalized by the amount of mHTT quantified by WB as shown in Supplementary Fig. S4. Mean values ± SD (1 σ) of n = 3 mice per group are shown.

We next tested if the polyQ length-dependent bias with MW1 detection Ab could be observed with the full length endogenous HTT protein using homogenates from striatum of 6 months old heterozygous HD-KI mice bearing different CAG repeat lengths in the *HTT* gene. Initially, MSD signal for mHTT was not observed to be polyQ length-dependent (Supplementary Fig. S4a). However, analysis of samples by western blot (WB) revealed a decreased amount of mHTT with increased polyQ length and for constant amount of total protein (Supplementary Fig. S4b). Normalization of MSD signal by the amount of mHTT quantified by WB confirmed the polyQ length-dependent correlation with MW1 detection Ab and full length endogenous HTT (R^2^ > 0.99; Fig. 2). It is remarkable to observe such similar correlation to what was seen with purified GST-FLAG-HTTexon1 using another method of normalization, demonstrating the robustness of our finding. A similar polyQ length correlation was observed independently of the capture Ab used (monoclonal rabbit EPR5526, targeting N-terminus of endogenous HTT protein or monoclonal rabbit D7F7, targeting middle region; Fig. 1a), confirming that only the avidity of MW1 detection Ab is involved (Fig. 2). Most striking, polyQ length-dependent bias for full length endogenous HTT was observed for a very large polyQ length range (from Q44 to Q188). All together, these observations show an inherent bias in mHTT detection by sandwich ELISA-based assays, which can be quantified and thus corrected.

### A novel method to evaluate polyQ length expansion in mHTT containing tissues using MSD assay

**Figure 3:**
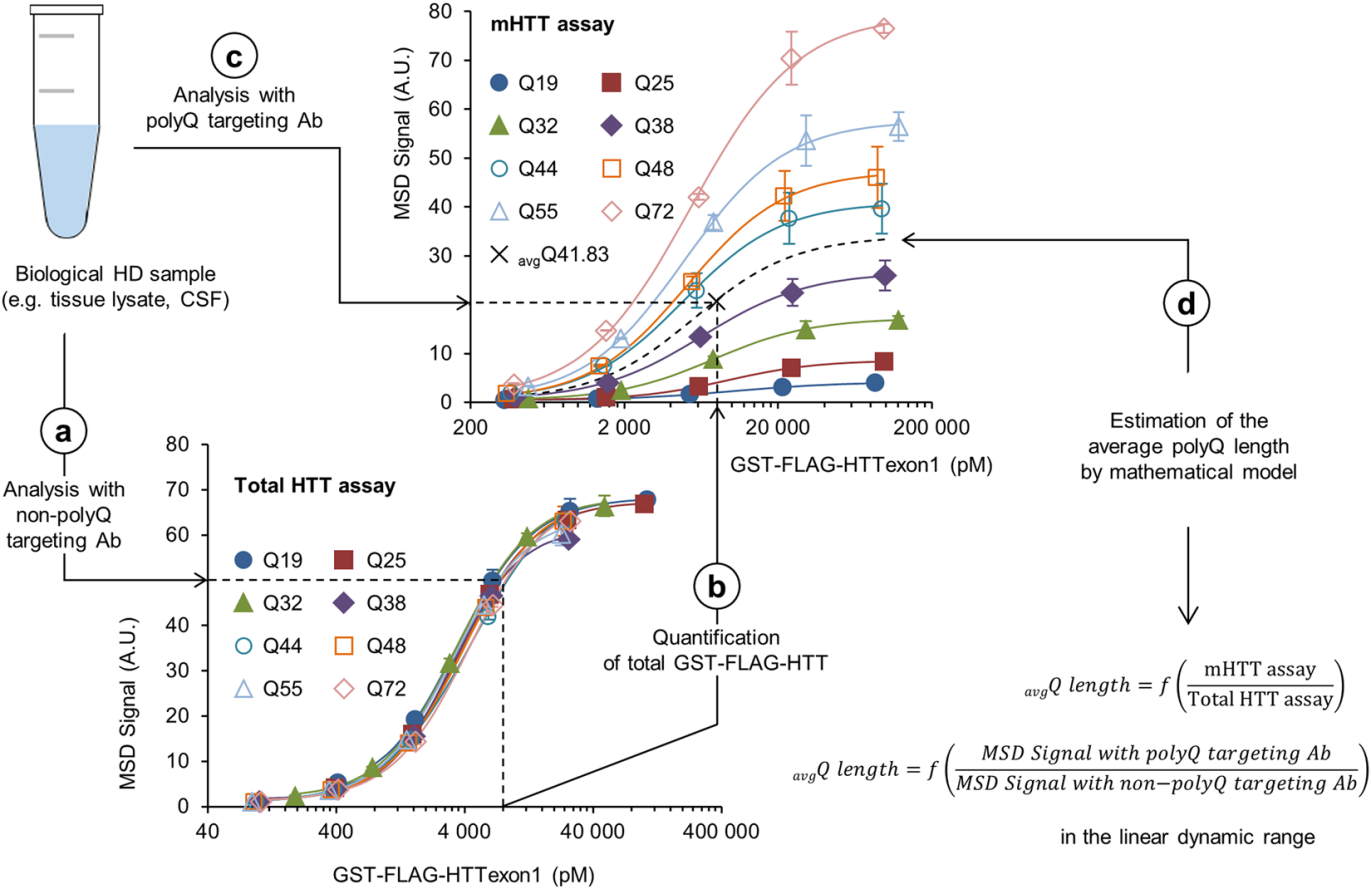
Method for HTT polyQ length quantification. HTT proteins exhibit a mosaicism of polyQ lengths in biological tissue prone to CAG repeat instability. To quantify average polyQ length in HTT proteins, the biological sample is quantified twice by sandwich ELISA-based assay with two pairs of Abs: one that includes a detection Ab that does not target the polyQ tract (**a**) to quantify total HTT (**b**) and another one that has a polyQ targeting detection Ab (**c**). This information is used in a mathematical model to determine the average polyQ length in HTT proteins (**d**) when samples are tested in the linear dynamic range.

We hypothesized that we could take advantage of polyQ length-dependent bias observed in mHTT detection by MSD assay to design a novel method for quantification of average polyQ length in a biological sample, such as tissue lysates or human biofluids (Fig. 3). In essence, we addressed if CAG repeat instability could be assessed at the protein level. The premises were 1) that HTT protein exhibits a mosaicism of polyQ lengths in biological tissue prone to CAG repeat instability^37–39^ and 2) that a population of HTT proteins with different polyQ lengths result in a similar detected signal to a single HTT protein with a polyQ length corresponding to the average polyQ length of the population. Briefly, the sample is analyzed twice by MSD assay: first, with non-polyQ targeting detection Ab such as MAB5492 that allows quantification of total HTT (WT and mutant form; Fig. 3a,b) then with polyQ targeting detection Ab that allows quantification of mHTT (Fig. 3c). Signal obtained in the linear dynamic range with polyQ targeting detection Ab for a determined HTT concentration can be used to estimate the average polyQ length by a mathematical model (Fig. 3d and Methods). Even if polyQ-targeting Abs preferentially bind expanded polyQ tract, they also interact, to a lower extent, with WT HTT. Similarly, Abs that do not target the polyQ tract interact with both WT and mHTT. Thus, our method which relies on quantification of both WT and mHTT, provides information on the average polyQ length in total HTT proteins.

**Figure 4:**
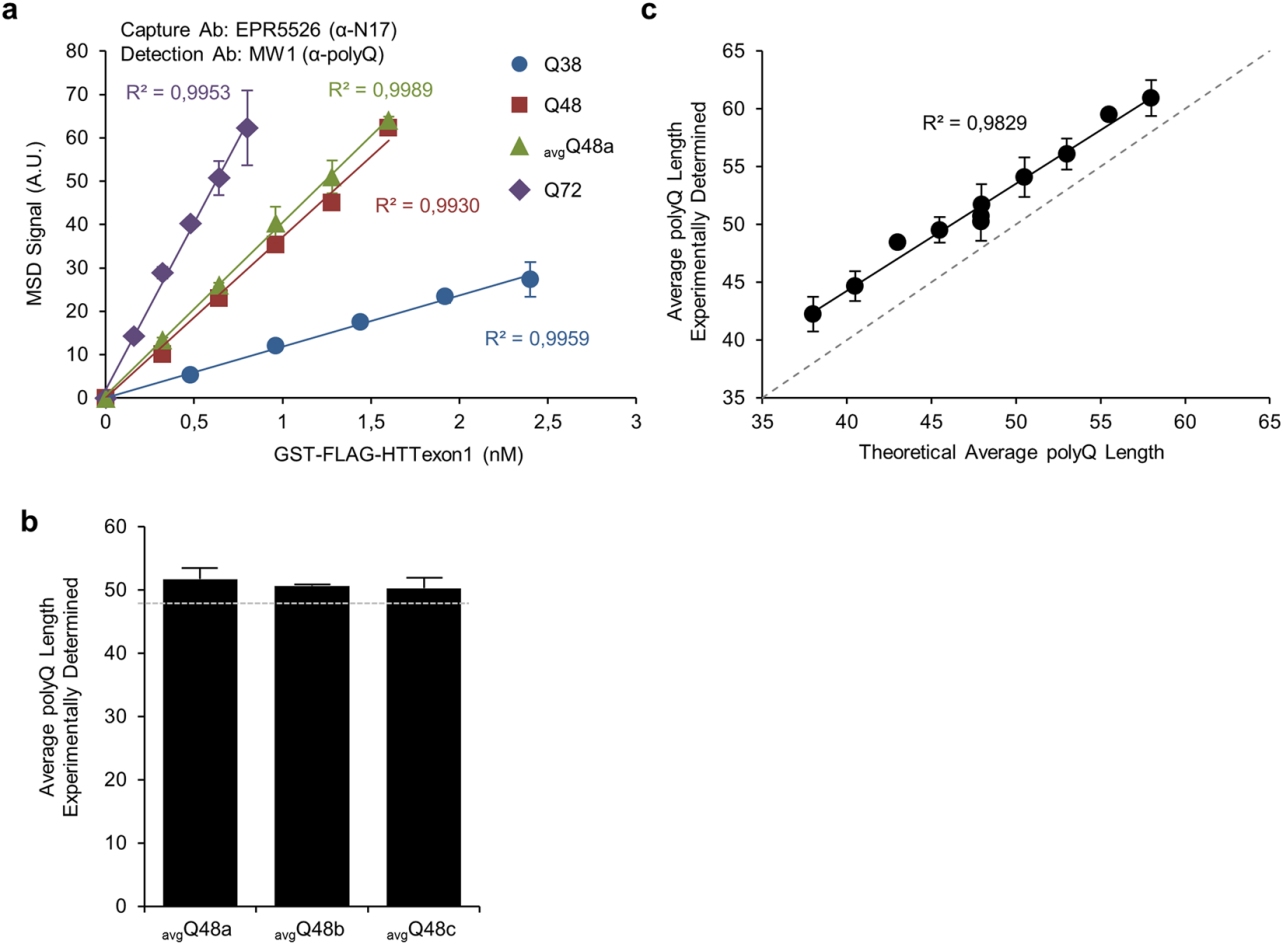
Pre-validation of method for average polyQ length quantification using MW1 and MAB5492 detection Abs: parallelism, dilution linearity, accuracy and robustness evaluation. (**a**) Serial dilution of a mix of GST-FLAG-HTTexon1 proteins with an average polyQ length of 48 residues (_avg_Q48a) gives similar results to a pure GST-FLAG-HTTexon1-Q48 using MW1 detection Ab. Serial dilution of pure GST-FLAG-HTTexon1-Q38 and -Q72 are also displayed for comparison. Mean values ± SD (1 σ) of duplicates of a single experiment are shown. (**b**) Average polyQ length experimentally determined for a similar average polyQ length of 48 residues done by different protein mixings. Dashed gray line corresponds to the theoretical average polyQ length. (**c)** Different average polyQ lengths experimentally determined are plotted as a function of theoretical average polyQ length. Dashed gray line corresponds to the perfect correlation between experimental and theoretical average polyQ lengths. Mean values ± propagated SD (1 σ) of duplicates of a single experiment are shown.

To mimic *in vitro* polyQ length mosaicism in HTT protein from biological tissue prone to CAG instability, different amounts of GST-FLAG-HTTexon1 proteins with variable polyQ lengths were mixed (Tables 1–3). Using polyQ targeting detection Ab MW1 and a mix of GST-FLAG-HTTexon1 proteins with an average polyQ length of 48 residues (_avg_Q48a), there was a similar MSD dose response to the standard curve obtained with pure GST-FLAG-HTTexon1-Q48 (Fig. 4a), indicating that the same average polyQ length could be determined at different concentrations of total GST-FLAG-HTTexon1 protein. The graph in Fig. 4a also displays results obtained with pure GST-FLAG-HTTexon1-Q38 and -Q72 for comparison. The average polyQ lengths experimentally determined did not exceed 13% of relative error, the highest relative error at the lowest concentration tested (Table 1). We then generated the same average polyQ length by different protein mixings of GST-FLAG-HTTexon1 (_avg_Q48a, _avg_Q48b and _avg_Q48c; Table 2). The average polyQ length experimentally determined at a single concentration was constant for a similar average polyQ length obtained by using different protein mixings (Fig. 4b and Table 2), highlighting the robustness of our method. Finally, we generated 9 different average polyQ lengths from _avg_Q38 to _avg_Q58 with 2.5Q increments (Table 3). The different average polyQ lengths determined experimentally at a single concentration exhibit a strong linear correlation with theoretical average polyQ lengths (R^2^ = 0.9829; Fig. 4c and Table 3). Intra-batch accuracy and precision for average polyQ length quantification were less than 13% of relative error and less than 4% of coefficient of variation for all conditions tested (Tables 1–3). Results obtained with other polyQ targeting Abs 1C2 and 3B5H10 were similar but with a lower accuracy (Supplementary Fig. S5 and Supplementary Tables S2–7). All together, these data validate the ability of our method to estimate the average polyQ length in a mix of HTT proteins with variable polyQ lengths, with the Ab pairs EPR5526-MW1 (for mHTT) and EPR5526-MAB5492 (for total HTT) being superior in accuracy.

**Table 1:** Quantification of the same average polyQ length (_avg_Q48a) at different protein concentrations with MW1 detection Ab.

Total protein concentration (pM)	Fraction of GST-FLAG-HTTexon1 mixed together					Average Q length experimentally determined	
	Q25	Q38	Q48	Q55	Q72	Mean ± SD (Cv %)	%RE
1,600	18%	21%	21%	20%	20%	48.44 ± 0.36 (0.75)	1.01
1,280	18%	21%	21%	20%	20%	48.29 ± 1.27 (2.63)	0.69
960	18%	21%	21%	20%	20%	51.01 ± 1.67 (3.28)	6.36
640	18%	21%	21%	20%	20%	51.14 ± 1.41 (2.76)	6.64
320	18%	21%	21%	20%	20%	53.90 ± 0.99 (1.83)	12.39

Average polyQ lengths experimentally determined by our method are expressed as mean values ± propagated SD (1 σ) of duplicates of a single experiment with their corresponding coefficient of variation (Cv %) and relative error (%RE) expressed as a percentage.

**Table 2:** Quantification of the same average polyQ length obtained by different protein mixings with MW1 detection Ab.

Theoretical Average Q length	Fraction of GST-FLAG-HTTexon1 mixed together					Average Q length experimentally determined	
	Q25	Q38	Q48	Q55	Q72	Mean ± SD (Cv %)	%RE
48a	18%	21%	21%	20%	20%	51.75 ± 1.75 (3.37)	7.90
48b	9%	20%	33%	30%	8%	50.71 ± 0.22 (0.44)	5.75
48c	12%	34%	18%	15%	21%	50.28 ± 1.68 (3.34)	4.90

Average polyQ lengths experimentally determined by our method are expressed as mean values ± propagated SD (1 σ) of duplicates of a single experiment with their corresponding coefficient of variation (Cv %) and relative error (%RE) expressed as a percentage.

**Table 3:** Quantification of different average polyQ lengths with MW1 detection Ab.

Theoretical Average Q length	Fraction of GST-FLAG-HTTexon1 mixed together					Average Q length experimentally determined	
	Q25	Q38	Q48	Q55	Q72	Mean ± SD (Cv %)	%RE
38	45%	21%	16%	11%	7%	42.26 ± 1.50 (3.55)	11.20
40.5	37%	21%	22%	10%	10%	44.69 ± 1.29 (2.89)	10.38
43	38%	14%	11%	22%	15%	48.48 ± 0.51 (1.05)	12.74
45.5	23%	25%	18%	17%	17%	49.54 ± 1.08 (2.19)	8.93
50.5	15%	15%	22%	24%	24%	54.08 ± 1.71 (3.17)	7.12
53	10%	22%	10%	26%	32%	56.09 ± 1.36 (2.43)	5.84
55.5	5%	13%	27%	19%	36%	59.52 ± 0.14 (0.23)	7.21
58	10%	5%	9%	32%	44%	60.94 ± 1.51 (2.53)	5.06

Average polyQ lengths experimentally determined by our method are expressed as mean values ± propagated SD (1 σ) of duplicates of a single experiment with their corresponding coefficient of variation (Cv %) and relative error (%RE) expressed as a percentage.

### Average polyQ length at the protein level correlates with average CAG repeat length at the DNA level in postmortem brain of HD mouse and HD patients

**Figure 5:**
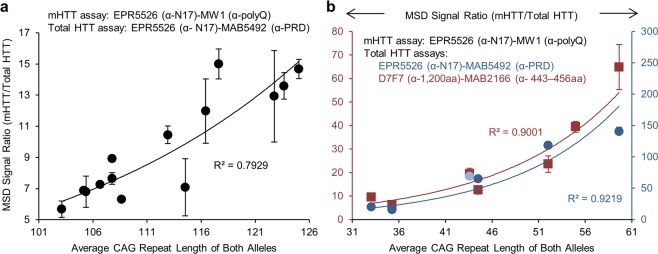
MSD signal ratio for mHTT by total HTT, corresponding to the average polyQ length, correlates with average CAG repeat length. (**a**) Striatal homogenates from 14 homozygous *Hdh*^*Q140*^ KI mice of different ages were analyzed by MSD assay for average polyQ length quantification (MSD signal ratio MW1/MAB5492 corresponding to mHTT/Total HTT). Results were plotted as a function of average CAG repeat length determined by PCR method in DNA extracted from the contralateral striatum of each animal (see Methods, Quantification of average CAG repeat length). It is unclear why there is more variability (larger SDs) in raw MSD signals for samples between ~108 and 124 CAG repeats than for other samples. All samples were processed at the same time and in the same manner, so it is likely that variation may be from pipetting. (**b**) Homogenates prepared from postmortem cortex of HD patients were analyzed by MSD assay for average polyQ length quantification (MSD signal ratio for mHTT by total HTT). Results were plotted as a function of average CAG repeat length determined by PCR method from the same sample lysates (see Methods, Quantification of average CAG repeat length). Light blue sample was below the level of detection (background + 3 SD) for total HTT assay and was not used for correlation. Mean values ± propagated SD (1 σ) of duplicates of a single experiment are shown. Please note that the MSD signal is normalized using the following MSD signal ratio (MSD of mHTT/MSD of Total HTT; plotted on y-axis in the figure). The MSD signal for total HTT is solely dependent on protein concentration and does not depend on polyQ length.

To establish whether our assay is suitable to measure the average polyQ length in endogenous HTT proteins from brain tissue, we examined striatum from homozygous *Hdh*^*Q140*^ KI mice from different litters and of different ages (from 3.3 to 13 mo). Since this mouse model was previously shown to exhibit intergenerational CAG repeat changes^40^, we expected to detect a variation in average polyQ length in HTT between animals. To test this idea, data obtained by MSD assay were compared with the extent of CAG repeat instability measured in gDNA from the contralateral striatum using PCR method adapted from Lee *et al*.^41^ (see Method section for details). The MSD signal is normalized using the MSD signal ratio (MSD of mHTT/MSD of Total HTT; plotted on y-axis in the figure). The MSD signal for total HTT is solely dependent on protein concentration and does not depend on polyQ length. Results showed that MSD signal ratios (EPR5526-MW1/EPR5526-MAB5492) obtained from striatum of HD mice exhibited a strong correlation with average CAG repeat length determined by PCR (R^2^ = 0.7929; Fig. 5a). Remarkably, the average CAG repeat length was determined from contralateral striatum which may have introduced some variation and could explain, at least in part, some outliers. Unfortunately, we could not interpolate the average polyQ length from these data because 1) the recombinant GST-FLAG-HTTexon1 proteins used as standards do not bear sufficient polyQ repeats tracts and 2) it was reported that the same concentration of the full length and truncated HTT proteins with similar polyQ lengths are detected with wide difference in intensity^31^. Even though we showed polyQ length correlation with full length endogenous mHTT (Fig. 2), anchor points of this correlation are probably different than those obtained with GST-FLAG-HTTexon1.

Having established that MSD signal ratios (EPR5526-MW1/EPR5526-MAB5492) for endogenous HTT could be correlated with CAG repeat length in HD mice, we next focused on analysis of human postmortem HD brain. We analyzed lysates from postmortem cortex of 2 adult and 5 juvenile HD (JHD) patients. Protein and DNA analysis were done in the same sample lysate for all samples. As it was shown that exon 1 of HTT is produced via incomplete splicing of the HTT pre-mRNA in HD patient tissue^42^, we used an additional Abs’ pair (D7F7-MAB2166) for total HTT quantification (WT + mutant form) that does not recognize the truncated form of HTT. The MSD signal ratios (mHTT/Total HTT) displayed a high correlation with average CAG repeat length determined by PCR for both Ab pairs used for normalization (R^2^ > 0.9; Fig. 5b) and a strong parallelism between them. Among the samples tested for total HTT quantification with EPR5526-MAB5492, two non-affected individuals were not used for correlation because their signals were below background signal for the level of detection (data not shown).

## Discussion

Currently, lowering mHTT is a major therapeutic strategy under investigation in many laboratories and in clinical trials for HD patients^43,44^, therefore accurate quantification using ultra-sensitive immunodetection methods is vital. mHTT can be preferentially distinguished from WT by polyQ targeting Abs^23–25^ sensitive to expanded polyQ repeats containing more epitopes than normal polyQ tracts. The increased avidity of such Abs for longer polyQ tracts was recognized as a potential bias in mHTT quantification^19,20,22,31^. However, even if the levels of mHTT were associated with inherited CAG repeat length^19,33–35^, polyQ length was considered as a minor contributor compared to mHTT protein concentration^20^. Previously, a series of purified truncated HTT proteins with different polyQ lengths was detected by TR-FRET assay and MW1 Ab^33^. The authors reported a 10- to 20-fold higher sensitivity for mHTT than WT HTT. They did not mention that with an increase of 7 glutamines, corresponding to the range of polyQs of HD patients tested in their study, they had a signal increase of ~40% for the same HTT protein concentration (see their Supplementary Fig. S1b). The analysis of large polyQ length series was not evaluated with sandwich-ELISA based methods currently used in clinical trial^20,31^. Here, we show that MSD signal detected with polyQ targeting Ab MW1 increases and, most of all, strongly correlates with polyQ length in purified N-terminal HTT fragments (Fig. 1d) and in endogenous full length HTT obtained from HD KI mice and human cortex (Figs. 2 and 5). Remarkably, this polyQ length dependent bias is evident for polyQ tracts that are in the range of adult onset HD patients as well as very large polyQ tracts (up to Q188). Our data suggest that even small polyQ length variations could lead to a large inaccuracy in mHTT quantification (Fig. 1e). When considering that somatic CAG repeat expansion occurs in HD brain^11,12^, the inaccuracy of mHTT quantification may be even greater.

Our findings raise questions about the reported increase in mHTT in CSF with disease progression using micro bead-based IP-flow cytometry and SMC assays^19,20,35^: is it solely due to mHTT concentration or might there be a contribution of CAG repeat instability? This is especially important if we consider that mHTT detected in CSF could preferentially come from dying cells exhibiting a very high level of instability. This issue is further complicated by findings that mHTT increases with disease progression in peripheral blood mononuclear cells (PBMC) but without significant difference in total HTT^33,34^. Initially, CAG repeat instability was proposed as a possible explanation for progressive increase in mHTT levels with no concomitant differences in total HTT level, but another likely explanation was a progressive accumulation of N-terminal fragments^33^. The latter explanation is challenged by a recent study showing no variation in N-terminal HTT level at different disease stages in PBMC^34^. The presence of CAG repeat instability is unlikely to influence the relative quantification of mHTT in current therapeutic silencing studies where a reduction in mHTT is measured as a change from baseline before treatment^21^, normalizing potential bias due to polyQ length difference between patients. Only CAG instability over the course of the longitudinal study could affect results.

In this study, we exploited the biasing effect of polyQ sensitive Abs in mHTT detection to design a novel method to assess the average polyQ length in HTT in samples where there is a population of HTT proteins with different polyQ lengths as might be expected under conditions of CAG repeat instability (Fig. 3). Our method relies on the normalization of MSD signal detected with polyQ targeting Ab MW1 by the amount of total HTT, corresponding to the MSD signal detected with non-polyQ targeting Ab MAB5492. The method proved to be sensitive, accurate and robust when tested using purified GST-FLAG-HTTexon1 (Fig. 4). Moreover, polyQ length assessment at the protein level strongly correlated with CAG repeat length at the DNA level in postmortem brain lysates from HD mice and patients (Fig. 5). It should be noted that for comparison of MSD signal ratio (mHTT/Total HTT), all samples were tested under the same conditions (same amount of total protein) and the detected signals were in the linear dynamic range of detection. Signal detected in 2 non-affected individuals was in the background signal and could not be used for MSD signal ratio and comparison with samples from HD individuals.

Studies have shown that the level of CAG repeat instability is higher in cortex than in cerebellum^10,11^. We confirmed this observation at the DNA level with our human sample set (data not shown). However, we were unable to obtain detectable signals for total HTT from cerebellar lysates, preventing the calculation of the MSD signal ratios. HTT protein was previously detected at lower levels in human cerebellum than in cortex of the same HD postmortem brains^37,45^. In our study, samples of human cerebellum and cortex were taken from the same brains and were processed in the same way and at the same time. Thus, we think the amount of total HTT present in cerebellar tissue is below the level of detection in our assay rather than an issue of the quality of the postmortem tissues or protein lysates.

Our method of determining average polyQ length relies on the correlation between MSD signal ratio (EPR5526-MW1/EPR5526-MAB5492) and polyQ length in HTT proteins. Using a different immuno-assay (AlphaLISA), different polyQ Ab (3B5H10) and non-polyQ (MAB2166 and D7F7) Abs and different materials (cell culture lysate and purified full length HTT), Baldo *et al*.^46^ reported that the ratio of mHTT/Total HTT signals increased with polyQ length. However, they did not perform further analysis. Our review of their data showed that similar to our findings, the ratio of their mHTT/Total HTT values shows a strong correlation with polyQ length (from their Fig. 4 and Fig. 5c; data not shown; R^2^ > 0.99).

Gold standard methods for determining CAG repeat instability involve PCR amplification from “bulk” or multiple small pools of genomic DNA. The negative correlation between CAG repeat length and PCR amplification efficiency represents a significant pitfall for accurate quantification^47,48^. However, despite a likely underestimation of CAG instability, especially for the bulk method that cannot detect the rare large expansions, the results obtained with these two methods exhibit a strong correlation^41^. Data obtained by bulk PCR in our studies exhibited a strong correlation with detected average polyQ length in HTT. Thus, we present a new complementary method to PCR for evaluating average instability at the protein level. Though less informative than PCR because it provides only average polyQ lengths without size distribution, it may allow an evaluation of expansion in tissues where HTT proteins, but not *HTT* gene, can be detected (e.g. CSF).

Our method of predicting HTT protein average instability relies on quantification of both WT and mHTT; both alleles must be expressed equally to correlate with CAG repeat length. We have observed that the level of mHTT decreases with polyQ length in HD-KI mouse models (Supplementary Fig. S4b). However in human, some western blot studies have observed an increased level of mHTT compared to WT in both adult and JHD brains^37^ or a lower amount of mHTT than WT solely in JHD brains^38^ and fibroblasts^38,49^. These inconsistent results could be due to a variety of factors including small sample size, the type of sample (brain or cell lines), the extent of separation of WT and mHTT or broader migration of mHTT in SDS-PAGE, probably due to CAG repeat instability and polyQ length mosaicism. Recently, a novel mass spectrometry-based method was developed to quantify allele specific HTT protein levels using polymorphic variants^50^. From the 28 adult HD subject-derived lymphoblast cell lines tested, levels of mHTT protein were highly associated with levels of WT HTT and were not correlated with the expanded CAG repeat size. These results argue against the idea that there is a potential effect of CAG repeat length on HTT protein level at least in the adult onset range. Although the impact of CAG repeat size on HTT expression levels in human brains remains largely unsolved, especially for JHD, our data, showing that polyQ length quantification significantly correlates with CAG repeat size, argues that both WT and mHTT levels are equal.

Our method relies on immunodetection of HTT proteins and therefore is subject to technical issues common to this approach, such as matrix influences or interfering substances. A fragment of HTT protein, corresponding to the 1–573 N-terminal aa, was reported to produce a higher signal than the full length HTT protein at comparable concentrations^31^. Fodale *et al*. consider results as a best estimate rather than absolute for mHTT quantification^31^. We were unable to obtain a series of stable purified full length HTT proteins with increasing polyQ lengths to compare to the results obtained with GST-FLAG-HTTexon1. The presence of HTT fragments has been reported in HD brain^51–53^. Additionally, flanking regions of the polyQ tract, which were sites used for total HTT detection in our assay, may be affected by polyQ length as described by others^54,55^ and may introduce a bias when determining Total HTT. It is noteworthy that the MSD signal ratios (mHTT/Total HTT) obtained from human cortical lysates with 2 different Abs’ pairs—targeting flanking polyQ regions and more C-terminal domains in HTT—displayed a high correlation with average CAG repeat length and a strong parallelism between them (Fig. 5b), suggesting that the contribution of truncated forms of HTT and the impact of polyQ length on flanking regions, if any, is negligible.

We have shown that MSD signal ratio (EPR5526-MW1/EPR5526-MAB5492) followed a simple polyQ length correlation in the linear dynamic range of our assay (Fig. 1d). We analyzed HD brain samples in this range of detection. The constraint of linear dynamic range could be a problem for polyQ assessment in samples with very low concentrations of HTT. However, results obtained with GST-FLAG-HTTexon1 showed that parameters from 4-parameters logistic regression are constant (Bottom and HillSlope) or strongly polyQ length dependent (Top and EC50) (Supplementary Fig. S6), allowing us to predict a regression curve for any polyQ length as illustrated in Fig. 3. Such an improved model for polyQ length assessment should overcome the limitation of our current study.

Genome-wide association studies identified potential genetic modifiers involved in CAG repeat instability^15,17^, opening an area for future therapeutic intervention. Our study represents a proof of principle for CAG repeat quantification at the protein level and paves the way for further studies. Our method relies on the detection of mHTT and Total HTT, which have both been detected and quantified in patient CSF using the SMC assay^19,20^, thus our assay potentially represents a way to study indirectly the extent of CAG repeat instability *in vivo* in the patient’s central nervous system. The lower limit of quantification of our MSD sandwich ELISA-based assay for mHTT (picomolar range) is not sensitive enough for quantification of mHTT in clinical CSF samples from HD patients. The SMC assay is required to reach femtomolar sensitivity^20,31,35^. Quantification of average CAG instability by our method adapted to SMC assay, could more accurately predict age of disease onset^12^ and be used in future clinical approaches that aim to reduce CAG repeat instability^14,56,57^.

## Methods

### Cloning

Plasmid vectors pGEX-6P-1 coding for GST-FLAG-HTTexon1 proteins with Q32, Q44 and Q55 were kindly provided by Erich Wanker^58^. DNA fragment coding for HTTexon 1 proteins with Q19 was available in-house^59^. DNA fragment coding for HTTexon 1 proteins with Q38 was a gift from Pamela Bjorkman^26^ (Addgene plasmid #11514). DNA fragments coding for HTTexon 1 proteins with Q25 and Q72 were kindly provided by Boxun Lu^60^. DNA fragment coding for HTTexon 1 proteins with Q48 was obtained by PCR from HD cell line (National Institute of Neurological Disorders and Stroke Repository at the Coriell Institute for Medical Research; catalog no. ND38551). DNA fragments coding for HTTexon1 were subcloned into the *EcoR*I and *Eag*I sites of pGEX-6P-1 vector (GE Healthcare Life Sciences), in frame with the GST-FLAG sequence. The coding regions of all vectors were verified by DNA sequencing.

### Protein production

GST-FLAG-HTTexon1 proteins were produced in E. coli BL21(DE3)pLysS competent cells (Thermo Fisher Scientific) grown at 16 °C in Lenox L broth base (ThermoFisherScientific) supplemented with ampicillin (100 μg/mL). For all proteins, the production was performed in 2 L flasks containing 400 mL of culture medium under constant agitation (220 rpm). Protein production was induced by adding IPTG (200 μM) when the optical density at 600 nm reached ~0.7. Bacteria were cultured overnight post-induction (~20 h), harvested by centrifugation and kept frozen (−20 °C).

### Protein purification

All purification steps were done on ice or at 4 °C. Lysis Buffer = Tris (10 mM; pH 8), NaCl (50 mM), KCl (50 mM), glycerol (10%). Elution Buffer = Tris (10 mM; pH 8), NaCl (150 mM), reduced glutathione (50 mM). Dialysis Buffer = phosphate (50 mM; pH 7.4). Bacterial pellets were thawed and suspended in 15 mL of lysis buffer containing lysozyme (10 mg/L), DTT (1 mM) and complete EDTA-free protease inhibitor cocktail (Roche). Bacteria were lysed by sonication during 2.5 min as follow: 3 s “on”, 10 s “off” using Sonic dismembrator model 500 set at 40% and 1/8” probe (Thermo Fisher Scientific). After centrifugation at 14,000 g for 1 h, the soluble bacterial extract was loaded at gravity flow on 400 μL of Glutathione Sepharose 4B affinity chromatography resin (GE Healthcare Life Sciences) in a Poly-Prep chromatography column (Bio-Rad). Resin was then washed with 10 volumes of lysis buffer, the first 5 volumes containing Triton detergent (0.5%) to improve the release of nonspecifically bound bacterial material. Finally, GST-FLAG-HTTexon1 proteins were sequentially eluted once with 100 μL then 5 times with 200 μL of elution buffer. Protein containing eluates (usually eluate 2 to 4) were diafiltrated by 5 washing out steps with dialysis buffer and Amicon Ultra-0.5 Centrifugal Filter Unit with Ultracel-3 membrane (MilliporeSigma). To avoid unnecessary losses upon freezing/thawing, protein stock concentrations were adjusted by diluting them in dialysis buffer and were stored at concentrations ranging from ~65 to ~100 μM. Comparison of concentrations before and after freeze/thawing showed negligible losses (<6%). To remove potential aggregates generated by the freezing/thawing process, thawed protein samples were centrifuged at 16,000 g and 4 °C for 5 min and the supernatant was collected. This centrifugation and supernatant collection step was performed twice. If used below 10 μM, bovine serum albumin (MilliporeSigma; #A2153) was added to proteins at 2 mg/mL to limit protein adsorption on pipette tips.

### Determination of purified protein concentration

Protein concentration was measured using its specific molar attenuation coefficient, after absorption spectrum scanning between 220 and 350 nm with DS-11 spectrophotometer (Denovix). Molar attenuation coefficient was computed with ProtParam tool on ExPASy bioinformatics resource portal^61^. Purity of full-length GST-FLAG-HTTexon1 proteins ranged from 67 to 90% depending on protein batch: a protein of the same size (~28 kDa) copurified with all proteins produced (Supplementary Fig. S1a), in proportion that is pure CAG repeat length dependent (Supplementary Fig. S1b). This product corresponded to the molecular mass of GST-FLAG and was detected with EPR5526 (anti-hHTT aa 1–100) but not with MW1 Ab by western blotting (Supplementary Fig. S1c). All together, these data suggest that 1) EPR5526 Ab targets HTT first 17 aa (N17), located N-terminally to the polyQ tract; 2) the 28 kDa species is composed of GST-FLAG-N17 and 3) only GST-FLAG-HTTexon1 protein can be detected by Ab pairs used for MSD assay. Quantification of protein concentration by absorbance at 280 nm, which measures absorbance of both GST-FLAG-HTTexon1 and GST-FLAG-N17 in solution, showed different results than Coomassie blue staining, which allowed a relative quantification of GST-FLAG-HTTexon1 (Supplementary Fig. S1d). To adjust protein concentration of GST-FLAG-HTTexon1 estimated by absorbance at 280 nm, correction factors for each batch of GST-FLAG-HTTexon1 protein were estimated based on relative quantification after SDS-PAGE (Supplementary Table S1). The same amount of GST-FLAG-HTTexon1 protein (according to absorbance at 280 nm) was fluorescently labeled with Amersham QuickStain Protein Labeling Kit (GE Healthcare) prior to SDS-PAGE. For each protein sample, fluorescent signal corresponding to GST-FLAG-HTTexon1 was quantified and normalized by average fluorescent signal of all GST-FLAG-HTTexon1 with different polyQ lengths. To validate experimentally these correction factors, we utilized the MSD platform for detection of GST-FLAG-HTTexon1 using EPR5526 capture Ab and MAB5492 detection Ab (Supplementary Fig. S2). Without correction of protein concentrations estimated by absorbance at 280 nm, the slope of standard curves obtained exhibited variations (Supplementary Fig. S2a) while after correction of protein concentrations, all standard curves overlap as expected (Supplementary Fig. S2b).

### Mouse and HD patient-derived material

Mice with human exon1 KI within the endogenous mouse *HTT* gene—*Hdh*^*Q50*^^62^, *Hdh*^*Q80*^^63^, *Hdh*^*Q111*^^64^, *Hdh*^*Q140*^^65^ and zQ175^66^—with the same strain background of C57BL/6 were obtained from The Jackson Laboratory and *Hdh*^*Q140*^ were bred and maintained at the MGH animal facility. Although named “Qn”, the average CAG repeat length of these mice is slightly different due to instability at the locus. Mice were anaesthetized with CO_2_ followed by cervical dislocation. The brain was rapidly removed, snap frozen using dry ice and stored at −80 °C for further use. After brain thawing to 4 °C, striatal tissues were dissected on ice, rapidly frozen using dry ice then stored at −80 °C for further use.

Human brain tissue was obtained from the Brain Tissue Resource Center (Belmont, MA), the University of Massachusetts, Department of Neuropathology and the Massachusetts General Hospital Neuropharmacology Laboratory Brain Bank. All tissue was quickly frozen and stored at −80 °C until further analysis. The time between death and brain dissection was variable but was always between 4 and 48 h. The dissections of neocortex and cerebellar cortex were performed to exclude the underlying white matter as much as possible.

Striatal and cerebellar samples were homogenized in buffer composed of HEPES (10 mM pH 7.4), sucrose (250 mM), EDTA (1 mM), complete EDTA-free protease inhibitor cocktail (Roche) and for some samples NaF (1 mM), Na_3_VO_4_ (1 mM) and were sonicated 10 s using Sonic dismembrator model 500 set at 20% and 1/8” probe (Thermo Fisher Scientific). Samples were then centrifuged at 16,000 g at 4 °C for 15 min. Supernatant was collected, aliquoted and stored at −80 °C for further use.

### Bradford assay

Protein Assay dye reagent (Bio-Rad) was diluted with 4 volumes of distilled, deionized water. One volume of biological sample (diluted 10- and 20-fold) or bovine serum albumin protein standard was mixed with 50 volumes of diluted dye reagent and 200 µL was loaded into 96-well plate (Thermo Fisher Scientific, #269620). Optical density at 600 nm was recorded with a Victor2 Multilabel plate reader (PerkinElmer). Total protein concentration was interpolated from bovine serum albumin standard curve made with 11 dilution points from 100 mg/mL and 2-fold dilution path. Protein concentration was expressed as mg/mL.

### SDS-PAGE electrophoresis

Three volumes of protein samples were mixed with one volume of NuPAGE™ LDS Sample Buffer 4 x (Thermo Fisher Scientific) and denatured by heating at 70 °C for 10 min. After a brief centrifugation step (1 min at 17,000 g), the same amount of denatured proteins was loaded on NuPAGE gel (Thermo Fisher Scientific) and separated by electrophoresis. Protein amounts were adjusted to optimize the different readouts: 90 pmol of GST-FLAG-HTTexon1 proteins for Coomassie blue staining; 3.75 pmol of GST-FLAG-HTTexon1 proteins for fluorescent detection; 15.6 pmol of GST-FLAG-HTTexon1 proteins for WB; 10 μg of total protein of mouse derived material. Purified proteins were migrated through 4–12% Bis-Tris gels with MES running buffer (Thermo Fisher Scientific) at 200 V constant and mouse brain lysates through 3–8% Tris-Acetate gels with Tris-Acetate running buffer (Thermo Fisher Scientific) at 120 V constant until suitable separation. Coomassie blue staining was done with PhastGel Blue R-350 (GE Healthcare Life Sciences) as recommended by the manufacturer and detected using white transillumination light of a FluorChem SP Imager (Alpha Innotech). Fluorescent labeling was done with Amersham QuickStain Protein Labeling kit (GE Healthcare Life Sciences) as follows: 1 μM of purified proteins were labeled with 0.25 μL of Cy5 dye reagent in a final volume of 12 μL of phosphate buffer 50 mM pH7.4 and incubated 30 min at RT. In-gel fluorescent detection was done using Odyssey Imaging System (LI-COR). Quantification was done using ImageJ software^67^.

### Antibodies

All antibodies used in this study are commercially available: EPR5526, rabbit monoclonal anti-HTT (Abcam Cat# ab109115, RRID:AB_10863082); MW1, mouse monoclonal anti-polyQ (DSHB Cat# mw1, RRID:AB_528290); 1C2, mouse monoclonal anti-polyQ (Millipore Cat# MAB1574, RRID:AB_94263); 3B5H10, mouse monoclonal anti-polyQ (Sigma-Aldrich Cat# P1874, RRID:AB_532270); MAB5492, mouse monoclonal anti-HTT (Millipore Cat# MAB5492, RRID:AB_347723); D7F7, rabbit monoclonal anti-HTT (Cell Signaling Technology Cat# 5656, RRID:AB_10827977); MAB2166, mouse monoclonal anti-HTT (Millipore Cat# MAB2166, RRID:AB_ 2123255); peroxidase-conjugated AffiniPure, donkey polyclonal anti-rabbit IgG (Jackson ImmunoResearch Labs Cat# 711–035–152, RRID:AB_10015282); peroxidase-conjugated AffiniPure, donkey polyclonal anti-mouse IgG (Jackson ImmunoResearch Labs Cat# 715–035–150, RRID:AB_2340770) and Sulfo-Tag labeled, goat polyclonal anti-mouse IgG (Meso Scale Discovery, Cat# R32AC, RRID:AB_2783819).

### Western blotting

Transfer to a nitrocellulose membrane was done using Trans-Blot® Turbo™ Transfer System according to manufacturer’s instructions. Membrane was then blocked with PBS-Tween 0.1% + 5% of Blotting-Grade Blocker (Bio-Rad) for 1 h on a rocking shaker. Membrane was then incubated overnight at 4 °C on a rocking shaker with 10 mL of EPR5526 (133.7 ng/mL; 1/10,000); MW1 (27.5 ng/mL; 1/10,000) or D7F7 Ab (1/2,000) in PBS-Tween 0.1% + 5% of Blotting-Grade Blocker. Membrane was then washed 3 × 10 min with 10 mL of PBS-Tween 0.1%. Membrane was then incubated for 1 h at RT on an orbital shaker with 10 mL of Peroxidase-conjugated AffiniPure Donkey anti-Mouse (1/5,000) or Peroxidase-conjugated AffiniPure Donkey anti-Rabbit IgG (1/2,500 or 1/5,000) in PBS-Tween 0.1% + 5% of Blotting-Grade Blocker. Membrane was then washed 3 × 10 min with 10 mL of PBS-Tween 0.1%. Acquisition was done using SuperSignal West Pico Chemiluminescent Substrate (Thermo Fisher Scientific) according to manufacturer’s instructions and FluorChem SP Imager (Alpha Innotech).

### MSD assay

Multi-Array 96-well standard plates (MSD) were coated overnight at 4 °C on a flat surface with 30 µL of D7F7 or EPR5526 capture Ab (2 µg/mL) in PBS pH 7.4 (Thermo Fisher Scientific). Plates were emptied and blocked with 150 µL of 3% bovine serum albumin (BSA) in PBS-Tween 0.05% pH 7.4 for 2 h at room temperature and 1,000 rpm on orbital microplate shaker (Scientific Industries). After 3 washes with 150 µL of washing buffer (PBS-Tween 0.05% pH 7.4), 30 µL of diluted samples were distributed into plates and incubated 1 h (for purified proteins) or 2 h (for biological samples) at room temperature and 1,000 rpm. The amount of biological material tested was adjusted for each pair of Abs to obtain signal in the linear dynamic range of detection: ~10 μg of total protein of mouse derived material (for D7F7-MW1 and EPR5526-MW1 mHTT assays); ~140 μg of total protein of mouse derived material (for EPR5526-MAB5492 Total HTT assay); 6 μg of total protein of human derived material for EPR5526-MW1 mHTT assays and 50 or 80 μg of total protein of human derived material for D7F7-MAB2166 or EPR5526-MAB5492 Total HTT assays respectively. Plates were then washed 3 times with washing buffer and incubated with 30 µL of detection antibody and incubated for 1 h at room temperature and 1,000 rpm. Depending on the type of sample, different concentrations of detection Abs were used for optimal signal-to-noise ratio: MW1 (2 µg/mL); 3B5H10 (2 µg/mL); 1C2 (1:1,000 or 1:2,500); MAB5492 (1:5,000 or 1:20,000) and MAB2166 (1:10,000). After 3 washes with 150 µL of washing buffer, 30 µL of goat anti-mouse SulfoTag secondary Ab (2 µg/mL) were distributed into plates and incubated 1 h at room temperature and 1,000 rpm. After 3 washes with 150 µL of washing buffer, 150 µL of 2X Read Buffer T with surfactant (MSD) were distributed into plates before reading on QuickPlex SQ120 instrument (MSD) according to manufacturer’s instructions.

### Regression analysis of MSD data

Calibration curves of purified proteins were fitted with a four-parameter logistic regression model, 1/Y^2^ weighting and least squares’ method using Solver, a Microsoft Excel Office 365 software add-in program. Four-parameter logistic regression model is:

1 $$MSD\,Signal=Bottom+\frac{{x}^{HillSlope}\times (Top-Bottom)}{{x}^{HillSlope}+EC{50}^{HillSlope}}$$

where *Bottom* and *Top* are plateaus of MSD signal; *x* is protein concentration; *EC50* is the protein concentration that gives MSD signal half way between *Bottom* and *Top*; *HillSlope* is a factor representing the steepness of the standard curve. Slope of standard curves in the linear dynamic range were determined as shown in Supplementary Data Set 1.

For other regression analysis, different models (linear, exponential, power, logarithmic and power) were tested with the least squares’ method using Microsoft Excel Office 365 software. Regression models with highest R-squared value were selected.

### Quantification of average polyQ length

When the amount of HTT is assessed in the linear dynamic range of our MSD assays, then:

2 $$MSD\,Signal=Concentration\,\times \,Slope$$

and we showed in Fig. 1d that:

3 $$polyQ\,Length=f(\frac{Slope\,(MW1)}{Slope\,(MAB5492)})$$

Combination of Eqs. (2) and (3) leads to:

4 $$polyQ\,Length=f(\frac{MSD\,Signal\,(MW1)}{MSD\,Signal(MAB5492)})$$

Both ratio of MSD signal obtained with MW1 by MSD signal obtained with MAB5492 or ratio of slope of MSD signal obtained with MW1 by ratio of slope of MSD signal obtained with MAB5492 could be used to quantify average polyQ length in biological samples. For MSD signal ratios or ratios of slope of MSD signals, propagation of error was calculated by the equation:

5 $$Propagated\,S{D}_{\frac{A}{B}}=\frac{A}{B}\times \sqrt{({(\frac{S{D}_{A}}{A})}^{2}+{(\frac{S{D}_{B}}{B})}^{2})}$$

PolyQ length was extrapolated from standard curve obtained by testing different concentrations of GST-FLAG-HTTexon1 with different polyQ lengths.

### PCR assay

Genomic DNA was isolated from tissues for somatic instability analysis using the DNeasy Blood & Tissue Kit (Qiagen). The size of the HTT CAG repeat was determined using a PCR assay that amplifies the HTT CAG repeat. The forward primer was fluorescently labeled with 6-FAM (Applied Biosystems) and products were resolved using the ABI 3730xl DNA analyzer (Applied Biosystems) with GeneScan 500 LIZ as internal size standard (Applied Biosystems).

### Quantification of average CAG repeat length

PCR amplification of trinucleotide repeats from tissue prone to CAG instability generates multiple PCR products, viewed using GeneMapper software as a cluster of peaks differing by a single CAG repeat unit^41^. The following steps were used to determine the average CAG repeat quantification: 1) WT and mutant huntingtin alleles were analyzed individually; 2) 5% (threshold factor) of the height of the highest peak was set as a relative peak height threshold (peaks with heights lower than this threshold level were excluded from quantification); 3) peak heights were normalized by dividing the peak height of each peak by the sum of the heights of all signal peaks; 4) the normalized peak heights were multiplied by their related CAG repeat lengths; 5) values from step 4 were summed to get the average CAG repeat length for each allele; 6) average CAG repeat length for each allele were averaged.

### Study approval

The animal protocol was approved by the MGH Subcommittee on Research Animal Care - Office of Laboratory Animal Welfare #2004N000248. All procedures conform to the USD Animal Welfare Act, “the Institute for Laboratory Animal Research Guide for the Care and Use of Laboratory Animals”, Physician Health Services Policy on Humane Care and Use of Laboratory Animals.

## Supplementary information

Supplementary info

Supplementary Dataset

## Data Availability

The datasets generated during and/or analysed during the current study are available from the corresponding author on reasonable request.

## References

[1] MacDonald ME (1993). A novel gene containing a trinucleotide repeat that is expanded and unstable on Huntington’s disease chromosomes. Cell.

[2] Nance MA, Mathias-Hagen V, Breningstall G, Wick MJ, McGlennen RC (1999). Analysis of a very large trinucleotide repeat in a patient with juvenile Huntington’s disease. Neurology.

[3] Lee J-M (2012). CAG repeat expansion in Huntington disease determines age at onset in a fully dominant fashion. Neurology.

[4] Djoussé L (2003). Interaction of normal and expanded CAG repeat sizes influences age at onset of Huntington disease. Am. J. Med. Genet. A..

[5] Wexler NS (2004). Venezuelan kindreds reveal that genetic and environmental factors modulate Huntington’s disease age of onset. Proc. Natl. Acad. Sci. USA.

[6] Caron, N. S., Wright, G. E. & Hayden, M. R. Huntington Disease. In *GeneReviews*® (eds Adam, M. P. *et al*.) (University of Washington, Seattle, 1993). 20301482

[7] Telenius H (1994). Somatic and gonadal mosaicism of the Huntington disease gene CAG repeat in brain and sperm. Nat. Genet..

[8] De Rooij KE, De Koning Gans PA, Roos RA, Van Ommen GJ, Den Dunnen JT (1995). Somatic expansion of the (CAG)n repeat in Huntington disease brains. Hum. Genet..

[9] Lee J-M, Pinto RM, Gillis T, St Claire JC, Wheeler VC (2011). Quantification of age-dependent somatic CAG repeat instability in Hdh CAG knock-in mice reveals different expansion dynamics in striatum and liver. PloS One.

[10] Kennedy L, Shelbourne PF (2000). Dramatic mutation instability in HD mouse striatum: does polyglutamine load contribute to cell-specific vulnerability in Huntington’s disease?. Hum. Mol. Genet..

[11] Kennedy L (2003). Dramatic tissue-specific mutation length increases are an early molecular event in Huntington disease pathogenesis. Hum. Mol. Genet..

[12] Swami M (2009). Somatic expansion of the Huntington’s disease CAG repeat in the brain is associated with an earlier age of disease onset. Hum. Mol. Genet..

[13] Dragileva E (2009). Intergenerational and striatal CAG repeat instability in Huntington’s disease knock-in mice involve different DNA repair genes. Neurobiol. Dis..

[14] Pinto RM (2013). Mismatch repair genes Mlh1 and Mlh3 modify CAG instability in Huntington’s disease mice: genome-wide and candidate approaches. PLoS Genet..

[15] Hensman Moss DJ (2017). Identification of genetic variants associated with Huntington’s disease progression: a genome-wide association study. Lancet Neurol..

[16] Consortium, G. M. of H. D. (GeM-H. *et al*. Huntington’s disease onset is determined by length of uninterrupted CAG, not encoded polyglutamine, and is modified by DNA maintenance mechanisms. *bioRxiv* 529768 10.1101/529768. (2019)

[17] Genetic Modifiers of Huntington’s Disease (GeM-HD) Consortium. (2015). Identification of Genetic Factors that Modify Clinical Onset of Huntington’s Disease. Cell.

[18] Lee J-M (2017). A modifier of Huntington’s disease onset at the MLH1 locus. Hum. Mol. Genet..

[19] Southwell AL (2015). Ultrasensitive measurement of huntingtin protein in cerebrospinal fluid demonstrates increase with Huntington disease stage and decrease following brain huntingtin suppression. Sci. Rep..

[20] Wild EJ (2015). Quantification of mutant huntingtin protein in cerebrospinal fluid from Huntington’s disease patients. J. Clin. Invest..

[21] Tabrizi SJ (2019). Targeting Huntingtin Expression in Patients with Huntington’s Disease. N. Engl. J. Med..

[22] Macdonald D (2014). Quantification assays for total and polyglutamine-expanded huntingtin proteins. PloS One.

[23] Ko J, Ou S, Patterson PH (2001). New anti-huntingtin monoclonal antibodies: implications for huntingtin conformation and its binding proteins. Brain Res. Bull..

[24] Trottier Y (1995). Polyglutamine expansion as a pathological epitope in Huntington’s disease and four dominant cerebellar ataxias. Nature.

[25] Miller J (2011). Identifying polyglutamine protein species *in situ* that best predict neurodegeneration. Nat. Chem. Biol..

[26] Bennett MJ (2002). A linear lattice model for polyglutamine in CAG-expansion diseases. Proc. Natl. Acad. Sci. USA.

[27] Li P (2007). The structure of a polyQ-anti-polyQ complex reveals binding according to a linear lattice model. Nat. Struct. Mol. Biol..

[28] Klein FAC (2007). Pathogenic and non-pathogenic polyglutamine tracts have similar structural properties: towards a length-dependent toxicity gradient. J. Mol. Biol..

[29] Klein FAC (2013). Linear and extended: a common polyglutamine conformation recognized by the three antibodies MW1, 1C2 and 3B5H10. Hum. Mol. Genet..

[30] Owens GE, New DM, West AP, Bjorkman PJ (2015). Anti-PolyQ Antibodies Recognize a Short PolyQ Stretch in Both Normal and Mutant Huntingtin Exon 1. J. Mol. Biol..

[31] Fodale V (2017). Validation of Ultrasensitive Mutant Huntingtin Detection in Human Cerebrospinal Fluid by Single Molecule Counting Immunoassay. J. Huntingt. Dis..

[32] Langbehn DR (2004). A new model for prediction of the age of onset and penetrance for Huntington’s disease based on CAG length. Clin. Genet..

[33] Weiss A (2012). Mutant huntingtin fragmentation in immune cells tracks Huntington’s disease progression. J. Clin. Invest..

[34] Hensman Moss DJ (2017). Quantification of huntingtin protein species in Huntington’s disease patient leukocytes using optimised electrochemiluminescence immunoassays. PloS One.

[35] Byrne Lauren M., Rodrigues Filipe B., Johnson Eileanor B., Wijeratne Peter A., De Vita Enrico, Alexander Daniel C., Palermo Giuseppe, Czech Christian, Schobel Scott, Scahill Rachael I., Heslegrave Amanda, Zetterberg Henrik, Wild Edward J. (2018). Evaluation of mutant huntingtin and neurofilament proteins as potential markers in Huntington’s disease. Science Translational Medicine.

[36] Dehay B, Weber C, Trottier Y, Bertolotti A (2007). Mapping of the epitope of monoclonal antibody 2B4 to the proline-rich region of human Huntingtin, a region critical for aggregation and toxicity. Biotechnol. J..

[37] Aronin N (1995). CAG expansion affects the expression of mutant Huntingtin in the Huntington’s disease brain. Neuron.

[38] Evers MM (2015). Making (anti-) sense out of huntingtin levels in Huntington disease. Mol. Neurodegener..

[39] Wheeler VC (2003). Mismatch repair gene Msh2 modifies the timing of early disease in Hdh(Q111) striatum. Hum. Mol. Genet..

[40] Neto JL (2017). Genetic Contributors to Intergenerational CAG Repeat Instability in Huntington’s Disease Knock-In Mice. Genetics.

[41] Lee J-M (2010). A novel approach to investigate tissue-specific trinucleotide repeat instability. BMC Syst. Biol..

[42] Neueder A (2017). The pathogenic exon 1 HTT protein is produced by incomplete splicing in Huntington’s disease patients. Sci. Rep..

[43] Wild EJ, Tabrizi SJ (2017). Therapies targeting DNA and RNA in Huntington’s disease. Lancet Neurol..

[44] Tabrizi SJ, Ghosh R, Leavitt BR (2019). Huntingtin Lowering Strategies for Disease Modification in Huntington’s Disease. Neuron.

[45] Schilling G (1995). Expression of the Huntington’s disease (IT15) protein product in HD patients. Hum. Mol. Genet..

[46] Baldo Barbara, Sajjad Muhammad Umar, Cheong Rachel Y., Bigarreau Julie, Vijayvargia Ravi, McLean Catriona, Perrier Anselme L., Seong Ihn Sik, Halliday Glenda, Petersén Åsa, Kirik Deniz (2018). Quantification of Total and Mutant Huntingtin Protein Levels in Biospecimens Using a Novel alphaLISA Assay. eneuro.

[47] Mutter GL, Boynton KA (1995). PCR bias in amplification of androgen receptor alleles, a trinucleotide repeat marker used in clonality studies. Nucleic Acids Res..

[48] Warner JP (1996). A general method for the detection of large CAG repeat expansions by fluorescent PCR. J. Med. Genet..

[49] Gutekunst CA (1995). Identification and localization of huntingtin in brain and human lymphoblastoid cell lines with anti-fusion protein antibodies. Proc. Natl. Acad. Sci. USA.

[50] Shin A (2017). Novel allele-specific quantification methods reveal no effects of adult onset CAG repeats on HTT mRNA and protein levels. Hum. Mol. Genet..

[51] DiFiglia M (1997). Aggregation of huntingtin in neuronal intranuclear inclusions and dystrophic neurites in brain. Science.

[52] Mende-Mueller LM, Toneff T, Hwang SR, Chesselet MF, Hook VY (2001). Tissue-specific proteolysis of Huntingtin (htt) in human brain: evidence of enhanced levels of N- and C-terminal htt fragments in Huntington’s disease striatum. J. Neurosci. Off. J. Soc. Neurosci..

[53] Kim YJ (2001). Caspase 3-cleaved N-terminal fragments of wild-type and mutant huntingtin are present in normal and Huntington’s disease brains, associate with membranes, and undergo calpain-dependent proteolysis. Proc. Natl. Acad. Sci. USA.

[54] Caron NS, Desmond CR, Xia J, Truant R (2013). Polyglutamine domain flexibility mediates the proximity between flanking sequences in huntingtin. Proc. Natl. Acad. Sci. USA.

[55] Daldin M (2017). Polyglutamine expansion affects huntingtin conformation in multiple Huntington’s disease models. Sci. Rep..

[56] Cinesi C, Aeschbach L, Yang B, Dion V (2016). Contracting CAG/CTG repeats using the CRISPR-Cas9 nickase. Nat. Commun..

[57] Suelves N, Kirkham-McCarthy L, Lahue RS, Ginés S (2017). A selective inhibitor of histone deacetylase 3 prevents cognitive deficits and suppresses striatal CAG repeat expansions in Huntington’s disease mice. Sci. Rep..

[58] Busch A (2003). Mutant huntingtin promotes the fibrillogenesis of wild-type huntingtin: a potential mechanism for loss of huntingtin function in Huntington’s disease. J. Biol. Chem..

[59] Kim M (1999). Mutant huntingtin expression in clonal striatal cells: dissociation of inclusion formation and neuronal survival by caspase inhibition. J. Neurosci. Off. J. Soc. Neurosci..

[60] Cui X (2014). TR-FRET assays of Huntingtin protein fragments reveal temperature and polyQ length-dependent conformational changes. Sci. Rep..

[61] Gasteiger Elisabeth, Hoogland Christine, Gattiker Alexandre, Duvaud S'everine, Wilkins Marc R., Appel Ron D., Bairoch Amos (2005). Protein Identification and Analysis Tools on the ExPASy Server. The Proteomics Protocols Handbook.

[62] White JK (1997). Huntingtin is required for neurogenesis and is not impaired by the Huntington’s disease CAG expansion. Nat. Genet..

[63] Shelbourne PF (1999). A Huntington’s disease CAG expansion at the murine Hdh locus is unstable and associated with behavioural abnormalities in mice. Hum. Mol. Genet..

[64] Wheeler VC (1999). Length-dependent gametic CAG repeat instability in the Huntington’s disease knock-in mouse. Hum. Mol. Genet..

[65] Menalled LB, Sison JD, Dragatsis I, Zeitlin S, Chesselet M-F (2003). Time course of early motor and neuropathological anomalies in a knock-in mouse model of Huntington’s disease with 140 CAG repeats. J. Comp. Neurol..

[66] Menalled LB (2012). Comprehensive behavioral and molecular characterization of a new knock-in mouse model of Huntington’s disease: zQ175. PloS One.

[67] Schneider CA, Rasband WS, Eliceiri KW (2012). NIH Image to ImageJ: 25 years of image analysis. Nat. Methods.

