# Supplementary material for: Assessing average somatic CAG repeat instability at the protein level
Source: Sci Rep. 2019 Dec 16;9:19152. doi: 10.1038/s41598-019-55202-x (PMC6915696; doi:10.1038/s41598-019-55202-x)
Supplement: Supplementary file 1 — Supplementary info [file 41598_2019_55202_MOESM1_ESM.pdf]

Manuscript SREP-19-35080

Assessing average somatic CAG repeat instability at the protein level.

Hubert Aviolat[1], Ricardo Mouro Pinto[2], Elizabeth Godschall[1], Ryan Murtha[2], Hannah E. Richey[1], Ellen Sapp[1], Petr Vodicka[1,3], Vanessa C. Wheeler[2], Kimberly B. Kegel-Gleason[1], and Marian DiFiglia[1]

[1]Department of Neurology, MassGeneral Institute for Neurodegenerative Disease, Massachusetts General Hospital, Harvard Medical School, Boston, MA, USA.

[2]Department of Neurology, Center for Genomic Medicine, Massachusetts General Hospital, Harvard Medical School, Boston, MA, USA.

[3]Present affiliation: Research Center PIGMOD, Institute of Animal Physiology and Genetics of the Czech Academy of Sciences, Libechov, Czech Republic.

Address correspondence to: Marian DiFiglia, MassGeneral Institute for Neurodegenerative Disease, 114 16th Street, Room 2002, Charlestown, MA 02129, USA. Phone: +1 617-726-8446; Email: difiglia@helix.mgh.harvard.edu.

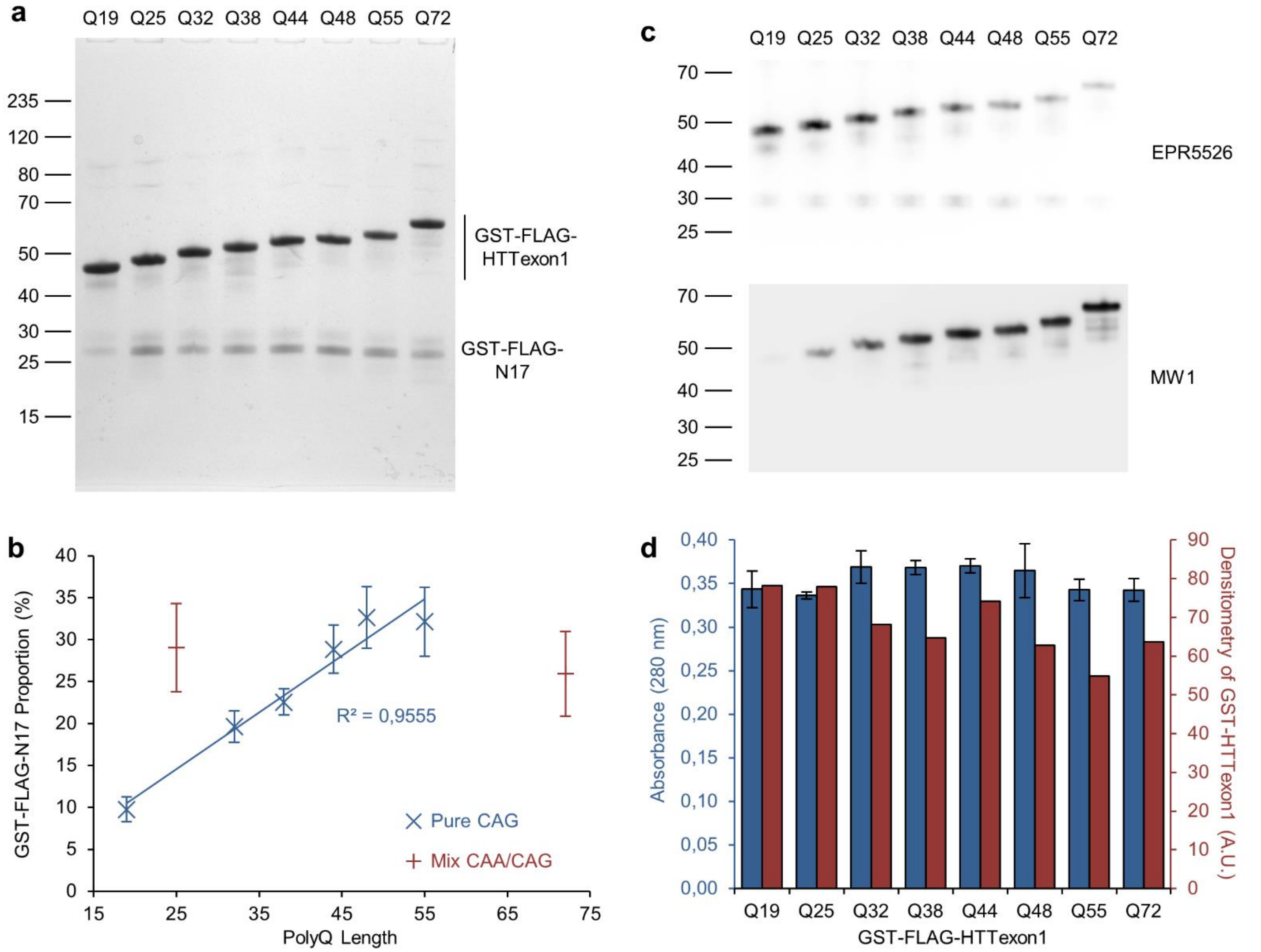

Supplementary Fig. S1 Protein production quality controls. Purity and quality control of recombinant GST-FLAG-HTTexon1 proteins revealed by Coomassie blue staining after SDS-PAGE (**a**) and by Western Blot (**c**). Primary Abs used are indicated on the right. Protein molecular weights on the left are expressed in kDa. **b** Proportion of GST-FLAG-N17 was assessed for each protein batch by Coomassie blue staining after SDS-PAGE. Mean values ± SD (1 σ) of n = 3 independent experiments are shown (R^2 = 0.9555). **d** Same amount of proteins, controlled by absorbance at 280 nm (blue columns), was loaded on the polyacrylamide gel shown in (**a**). Densitometry of GST-FLAG-HTTexon1 proteins (red columns) shows discrepancy with absorbance at 280 nm. For absorbance, mean values ± SD (1 σ) of triplicates are shown. Full-length Western Blots are shown on pages 16 and 17 of Supplementary Information file.

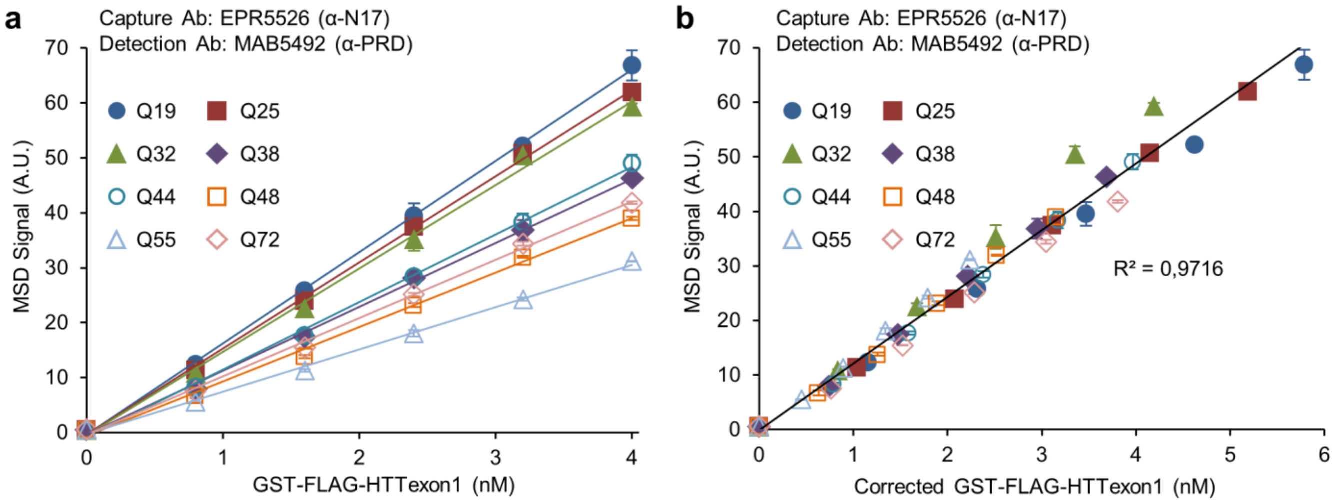

Supplementary Fig. S2 Adjustment of GST-FLAG-HTTexon1 concentration, estimated by absorbance at 280 nm in presence of GST-FLAG-N17, by correction factor. **a** Calibration curve performance for GST-FLAG-HTTexon1 protein using MAB5492 detection Ab. **b** Replotted data shown in (**a**) using corrected protein concentrations shows high correlation (R^2 = 0.9716). Mean values ± SD (1 σ) of duplicates of a single experiment are shown. A.U. is artificial units.

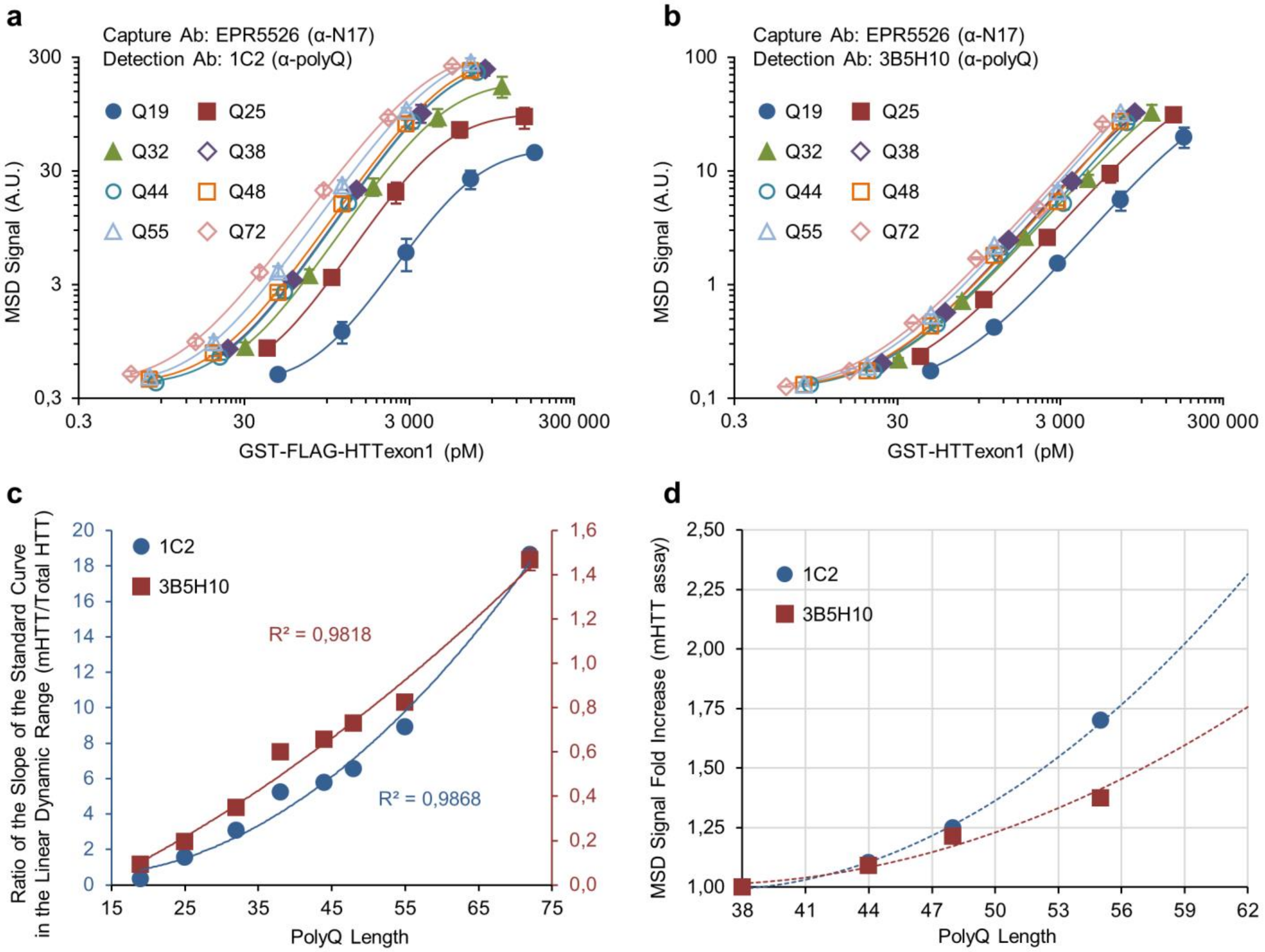

Supplementary Fig. S3 HTT quantification bias by MSD assay using 1C2 and 3B5H10 polyQ targeting detection Abs. **a, b** Calibration curve performance for GST-FLAG-HTTexon1 protein using 1C2 (**a**) and 3B5H10 (**b**) detection Abs. Curves were fitted with a four-parameter logistic regression model with $1/Y^2$ weighting. Mean values ± SD (1 σ) of duplicates of a single experiment are shown. **c** Plot of ratio of the slopes determined from standard curves in the linear dynamic range shown in (**a**) and (**b**) as a function of polyQ length exhibits strong correlation ($R^2 > 0.98$). Mean values ± propagated SD (1 σ) of duplicates of a single experiment are shown. **d** Using the polyQ length-dependent correlations shown in (**c**), MSD signal increases as a function of polyQ length with a constant amount of mHTT protein were extrapolated for both 1C2 and 3B5H10 detection Abs. PolyQ lengths ranging from Q38 to Q62 correspond to the polyQ length range seen in adult HD patients.

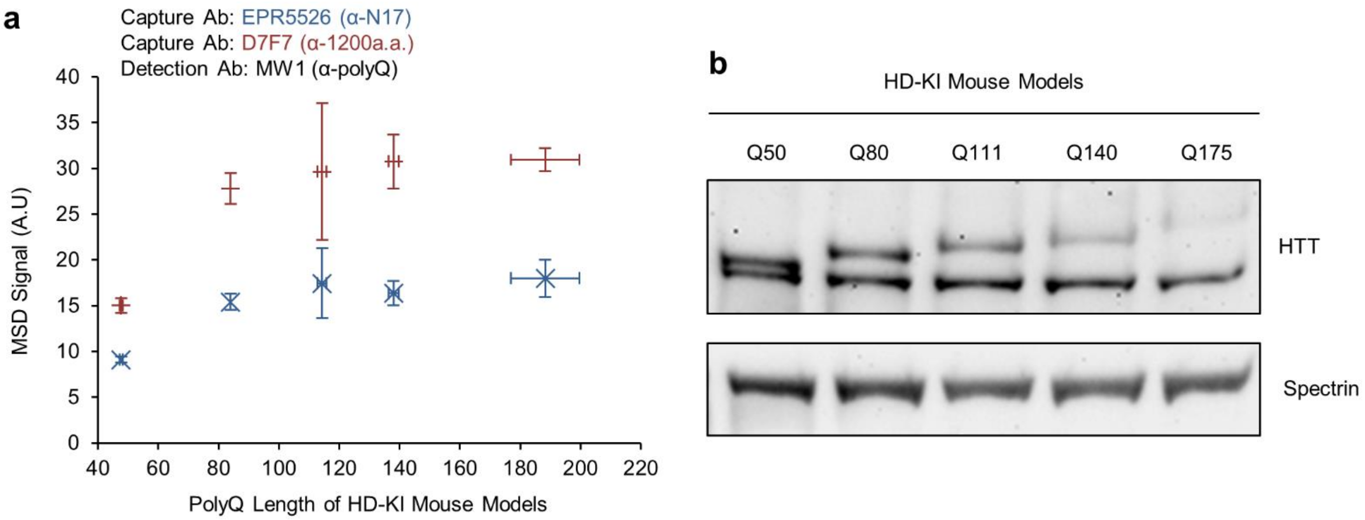

Supplementary Fig. S4 MSD signal normalization of HD-KI mouse samples. **a** Homogenates from striatum of heterozygous HD-KI mice with different inherited CAG repeats in the HD allele were analyzed by MSD assay for detection of mHTT with two different capture Abs (EPR5526 and D7F7) and MW1 detection Ab. Mean values ± SD (1 σ) of n = 3 mice per group are shown. **b** The same homogenates from striatum of HD-KI mice were analyzed by WB for detection of HTT with D7F7 Ab and mHTT was quantified by densitometry for each HD-KI mouse model. Results in Figure 2 are MSD signals shown in (**a**) normalized by the amount of mHTT quantified in (**b**). Full-length Western Blots are shown on pages 18 and 19 of Supplementary Information file.

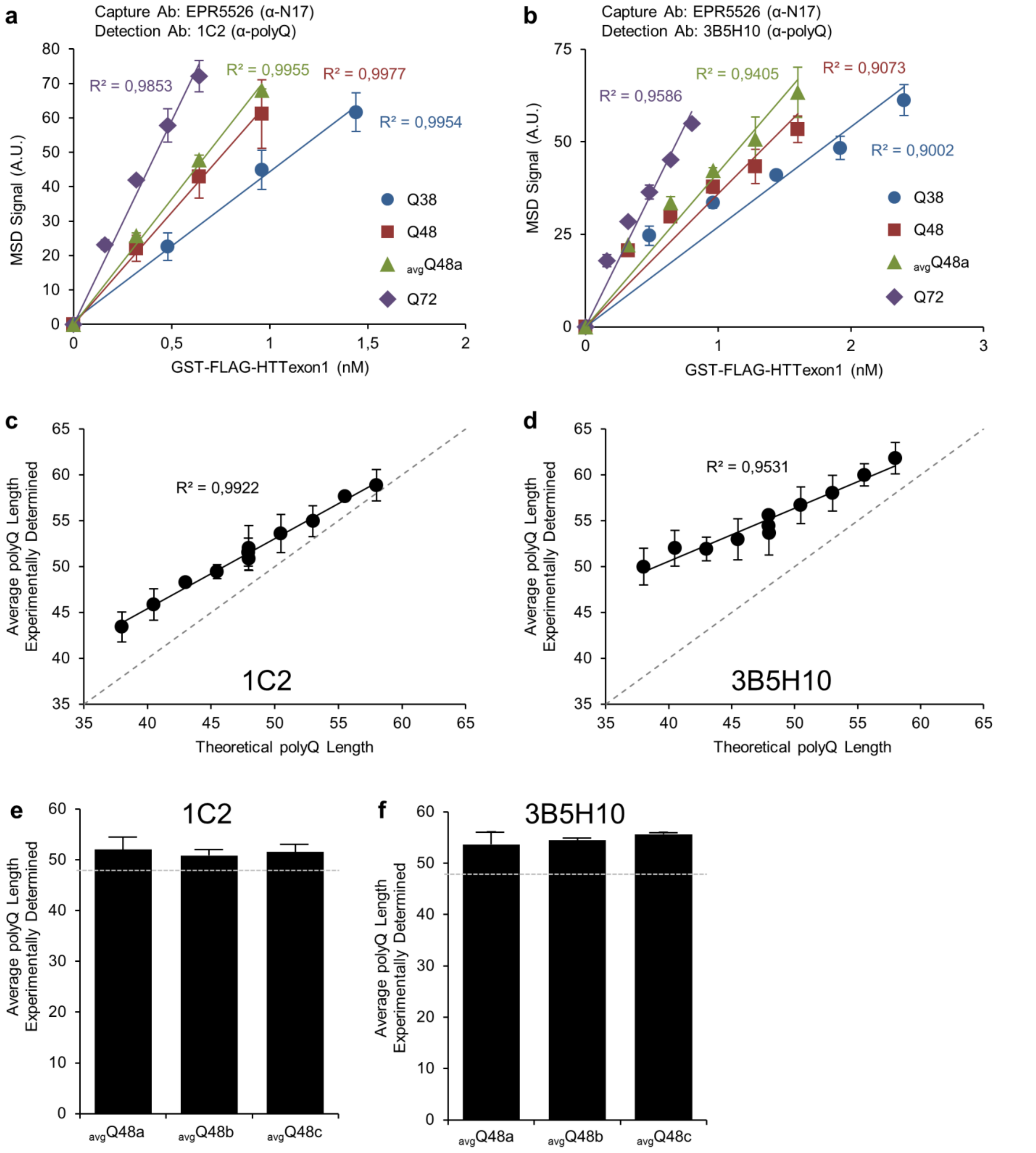

Supplementary Fig. S5 Pre-validation of method for average polyQ length quantification using 1C2, 3B5H10 and MAB5492 detection Abs. **a, b** Serial dilution of a mix of GST-FLAG-HTTexon1 proteins with an average polyQ length of 48 residues ($_{avg}$Q48a) gives similar results to pure GST-FLAG-HTTexon1-Q48 using 1C2 (**a**) or 3B5H10 (**b**) detection Abs. Serial dilution of pure GST-FLAG-HTTexon1-Q38 and -Q72 are also displayed for comparison. Mean values ± SD (1 σ) of duplicates of a single experiment are shown. **c, d** Different average polyQ lengths experimentally determined are plotted as a function of theoretical average polyQ length for both 1C2 (R^2 = 0.9922) (**c**) and 3B5H10 (R^2 = 0.9531) (**d**) detection Abs. Dashed gray line corresponds to the

perfect correlation between experimental and theoretical average polyQ lengths. **e, f** Average polyQ length experimentally determined for a similar average polyQ length of 48 residues done by different protein mixings with both 1C2 (**e**) and 3B5H10 (**f**). Dashed gray line corresponds to the theoretical average polyQ length. Mean values ± propagated SD (1 σ) of duplicates of a single experiment are shown.

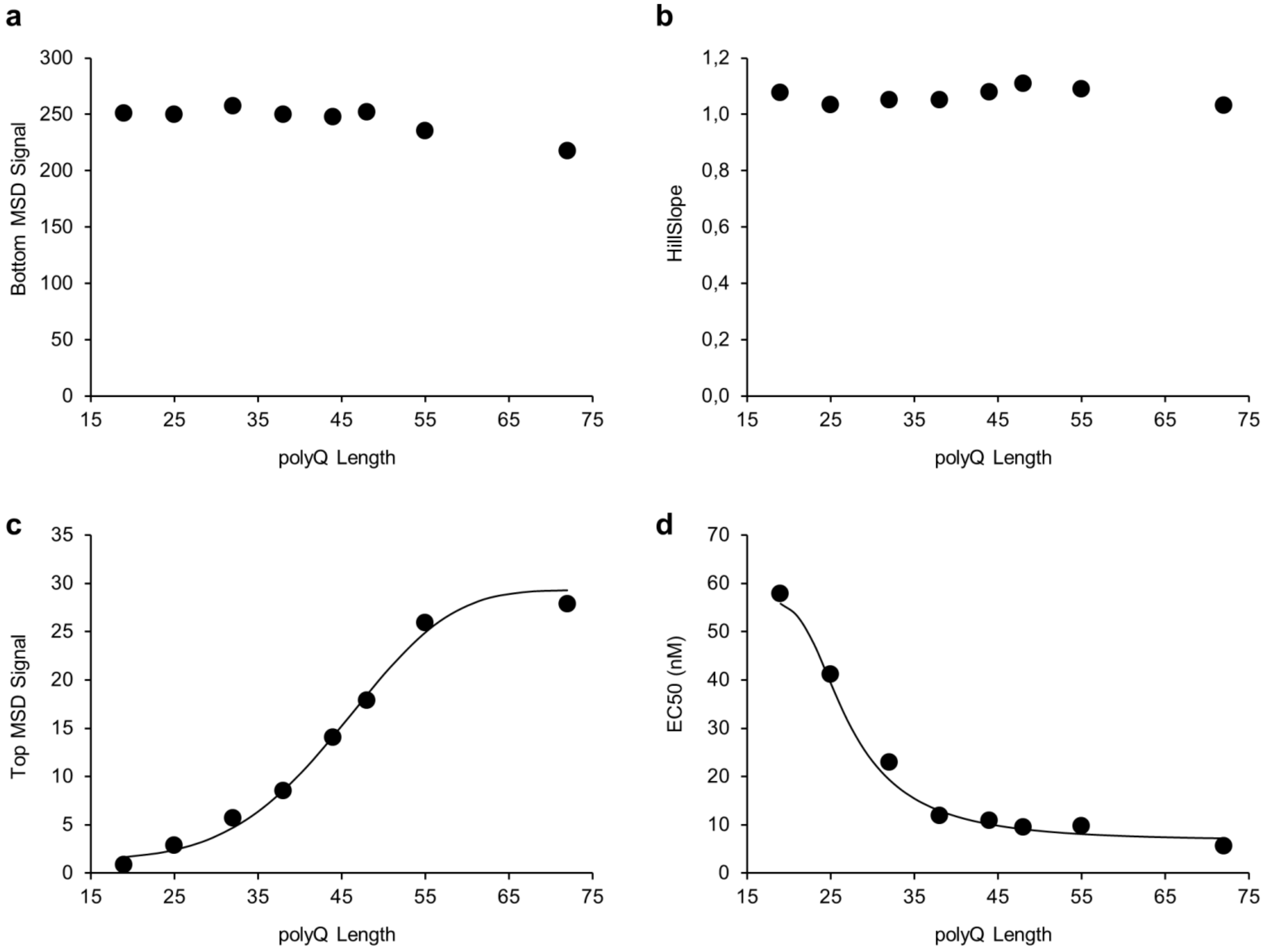

Supplementary Fig. S6 Parameters of 4-parameter logistic regression for mHTT are constant or polyQ length dependent. Results obtained with GST-FLAG-HTTexon1 in Figure 1B showed that parameters from 4-parameters logistic regression (detailed in Methods) are constant – Bottom (**a**) and HillSlope (**b**) parameter – or strongly polyQ length dependent – Top (**c**) and EC50 (**d**) parameter. Curves were fitted with a five-parameter logistic regression model without weighting.

PolyQ Length	Mean	SD	Cv (%)
19	1.446	0.112	7.774
25	1.296	0.057	4.385
32	1.047	0.198	18.904
38	0.922	0.132	14.343
44	0.991	0.065	6.563
48	0.788	0.072	9.173
55	0.559	0.125	22.298
72	0.951	0.087	9.125

Supplementary Table S1. Protein concentration correction factors.
Mean values of n = 5 independent experiments are shown. Cv = coefficient of variation expressed as percentage.

Total protein concentration (pM)	Fraction of GST-FLAG-HTTexon1 mixed together					Average Q length experimentally determined	
	Q25	Q38	Q48	Q55	Q72	Mean ± SD (Cv %)	%RE
1,600	18%	21%	21%	20%	20%	44.02 ± 1.48 (3.36)	-8.22
1,280	18%	21%	21%	20%	20%	45.44 ± 1.48 (3.26)	-5.26
960	18%	21%	21%	20%	20%	49.76 ± 0.80 (1.61)	3.76
640	18%	21%	21%	20%	20%	51.84 ± 1.98 (3.82)	8.09
320	18%	21%	21%	20%	20%	56.77 ± 1.20 (2.11)	18.37

Supplementary Table S2. Quantification of the same average polyQ length ($_{avg}$Q48a) at different protein concentrations with 1C2 detection Ab.
Average polyQ lengths experimentally determined by our method are expressed as mean values ± propagated SD (1 σ) of duplicates of a single experiment with their corresponding coefficient of variation (Cv %) and relative error (%RE) expressed as a percentage.

Theoretical Average Q length	Fraction of GST-FLAG-HTTexon1 mixed together					Average Q length experimentally determined	
	Q25	Q38	Q48	Q55	Q72	Mean ± SD (Cv %)	%RE
48a	18%	21%	21%	20%	20%	52.03 ± 2.44 (4.69)	8.49
48b	9%	20%	33%	30%	8%	50.84 ± 1.20 (2.37)	6.03
48c	12%	34%	18%	15%	21%	51.56 ± 1.53 (2.96)	7.57

Supplementary Table S3. Quantification of the same average polyQ length obtained by different protein mixings with 1C2 detection Ab.
Average polyQ lengths experimentally determined by our method and 1C2 detection Ab are expressed as mean values ± propagated SD (1 σ) of duplicates of a single experiment with their corresponding coefficient of variation (Cv %) and relative error (%RE) expressed as a percentage.

Theoretical Average Q length	Fraction of GST-FLAG-HTTexon1 mixed together					Average Q length experimentally determined	
	Q25	Q38	Q48	Q55	Q72	Mean ± SD (Cv %)	%RE
38	45%	21%	16%	11%	7%	43.42 ± 1.65 (3.79)	14.26
40.5	37%	21%	22%	10%	10%	45.86 ± 1.69 (3.68)	13.26
43	38%	14%	11%	22%	15%	48.30 ± 0.51 (1.05)	12.32
45.5	23%	25%	18%	17%	17%	49.46 ± 0.76 (1.53)	8.75
50.5	15%	15%	22%	24%	24%	53.59 ± 2.07 (3.86)	6.13
53	10%	22%	10%	26%	32%	54.95 ± 1.70 (3.10)	3.67
55.5	5%	13%	27%	19%	36%	57.66 ± 0.28 (0.49)	3.86
58	10%	5%	9%	32%	44%	58.88 ± 1.70 (2.89)	1.52

Supplementary Table S4. Quantification of different average polyQ lengths with 1C2 detection Ab.

Average polyQ lengths experimentally determined by our method and 1C2 detection Ab are expressed as mean values ± propagated SD (1 σ) of duplicates of a single experiment with their corresponding coefficient of variation (Cv %) and relative error (%RE) expressed as a percentage.

Total protein concentration (pM)	Fraction of GST-FLAG-HTTexon1 mixed together					Average Q length experimentally determined	
	Q25	Q38	Q48	Q55	Q72	Mean ± SD (Cv %)	%RE
1,600	18%	21%	21%	20%	20%	46.12 ± 2.16 (4.69)	-3.85
1,280	18%	21%	21%	20%	20%	46.03 ± 2.44 (5.30)	-4.03
960	18%	21%	21%	20%	20%	50.61 ± 0.89 (1.77)	5.52
640	18%	21%	21%	20%	20%	55.55 ± 2.28 (4.10)	15.83
320	18%	21%	21%	20%	20%	66.40 ± 1.04 (1.56)	38.44

Supplementary Table S5. Quantification of the same average polyQ length ($_{avg}$Q48a) at different protein concentrations with 3B5H10 detection Ab.
Average polyQ lengths experimentally determined by our method are expressed as mean values ± propagated SD (1 σ) of duplicates of a single experiment with their corresponding coefficient of variation (Cv %) and relative error (%RE) expressed as a percentage.

Theoretical Average Q length	Fraction of GST-FLAG-HTTexon1 mixed together					Average Q length experimentally determined	
	Q25	Q38	Q48	Q55	Q72	Mean ± SD (Cv %)	%RE
48a	18%	21%	21%	20%	20%	53.67 ± 2.43 (4.53)	11.90
48b	9%	20%	33%	30%	8%	54.44 ± 0.49 (0.90)	13.53
48c	12%	34%	18%	15%	21%	55.61 ± 0.41 (0.73)	16.03

Supplementary Table S6. Quantification of the same average polyQ length obtained by different protein mixings with 3B5H10 detection Ab.
Average polyQ lengths experimentally determined by our method are expressed as mean values ± propagated SD (1 σ) of duplicates of a single experiment with their corresponding coefficient of variation (Cv %) and relative error (%RE) expressed as a percentage.

Theoretical Average Q length	Fraction of GST-FLAG-HTTexon1 mixed together					Average Q length experimentally determined	
	Q25	Q38	Q48	Q55	Q72	Mean ± SD (Cv %)	%RE
38	45%	21%	16%	11%	7%	49.97 ± 2.00 (4.01)	31.51
40.5	37%	21%	22%	10%	10%	52.00 ± 1.93 (3.72)	28.42
43	38%	14%	11%	22%	15%	51.93 ± 1.30 (2.51)	20.76
45.5	23%	25%	18%	17%	17%	52.98 ± 2.23 (4.20)	16.50
50.5	15%	15%	22%	24%	24%	56.69 ± 2.00 (3.53)	12.29
53	10%	22%	10%	26%	32%	58.00 ± 1.95 (3.37)	9.43
55.5	5%	13%	27%	19%	36%	60.00 ± 1.23 (2.05)	8.06
58	10%	5%	9%	32%	44%	61.80 ± 1.71 (2.76)	6.56

Supplementary Table S7. Quantification of different average polyQ lengths with 3B5H10 detection Ab.
Average polyQ lengths experimentally determined by our method are expressed as mean values ± propagated SD (1 σ) of duplicates of a single experiment with their corresponding coefficient of variation (Cv %) and relative error (%RE) expressed as a percentage.

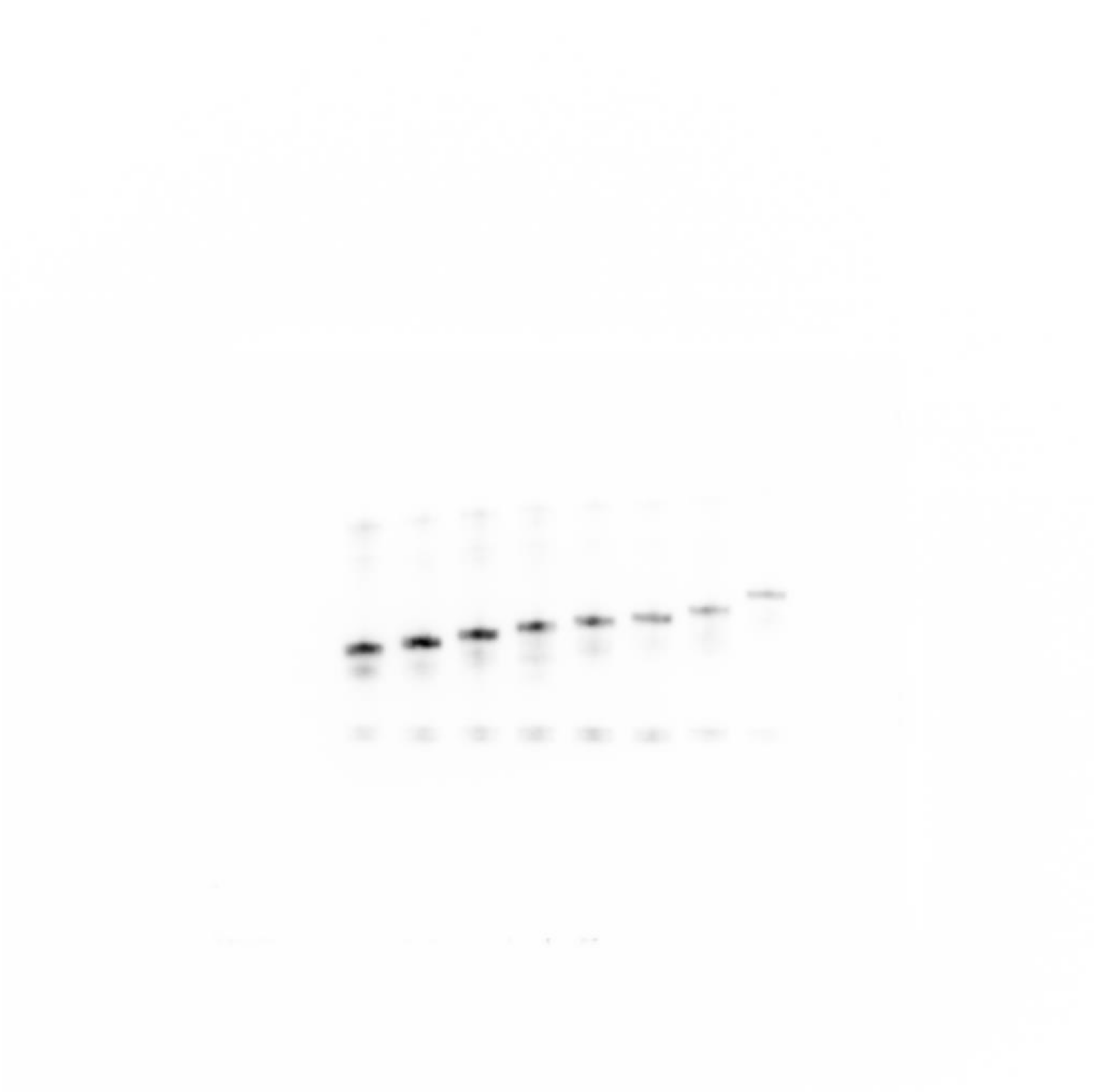

Supplementary Fig. 1c (upper panel)

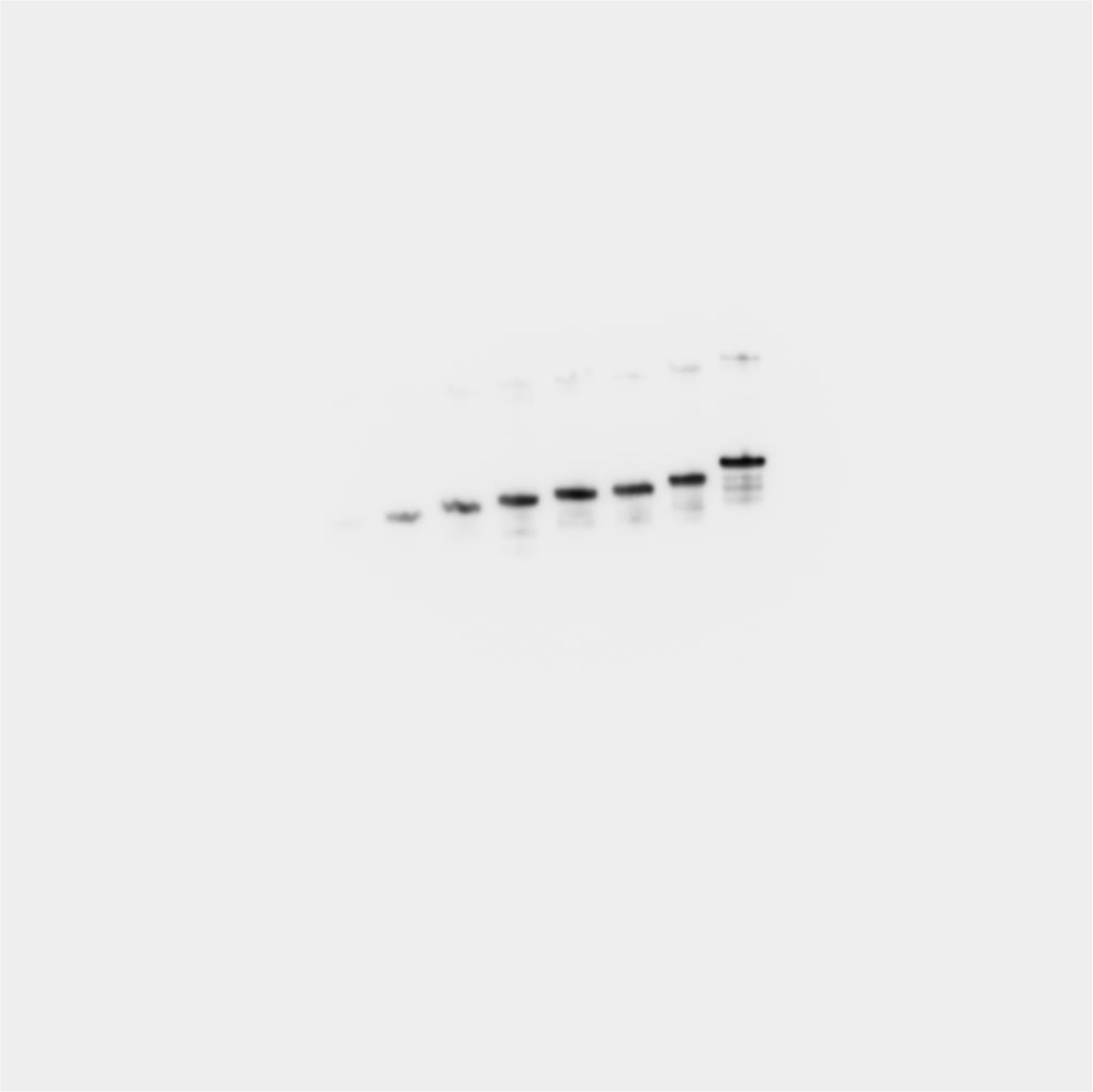

Supplementary Fig.1c (lower panel)

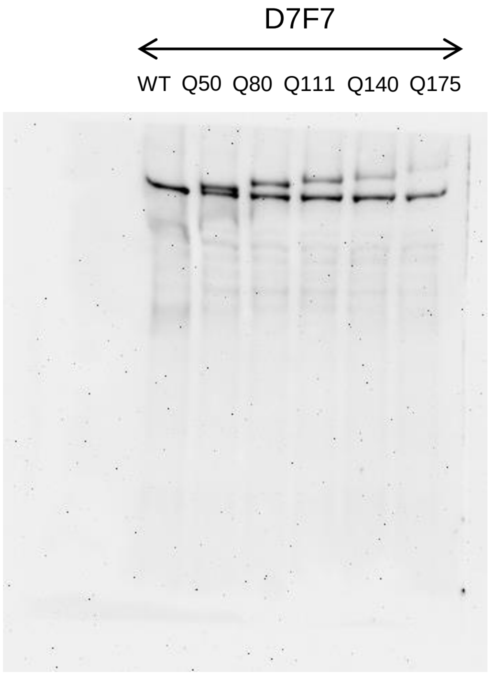

Supplementary Fig.4b (upper panel)

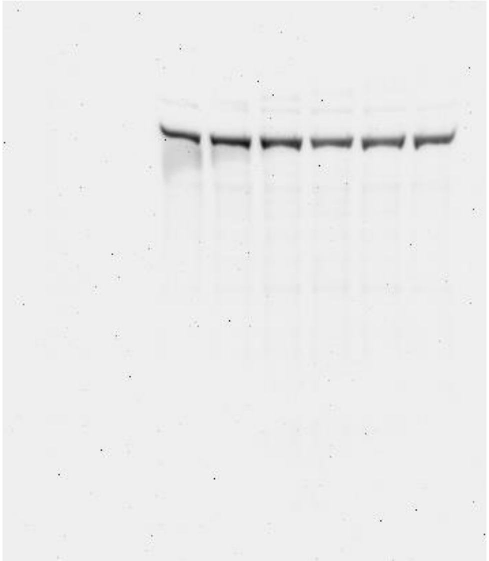

Supplementary Fig.4b (lower panel)
